# Cloning and functional analysis of the *FAD2* gene family from desert shrub *Artemisia sphaerocephala*

**DOI:** 10.1186/s12870-019-2083-5

**Published:** 2019-11-08

**Authors:** Xiumei Miao, Lijing Zhang, Xiaowei Hu, Shuzhen Nan, Xiaolong Chen, Hua Fu

**Affiliations:** State Key Laboratory of Grassland Agro-ecosystems; Key Laboratory of Grassland Livestock Industry Innovation; Ministry of Agriculture and Rural Affairs; Engineering Research Center of Grassland Industry, Ministry of Education; College of Pastoral Agriculture Science and Technology, Lanzhou University, Lanzhou, 730020 People’s Republic of China

**Keywords:** Fatty acid desaturase, linoleic acid, expression analysis, subcellular localization, yeast expression

## Abstract

**Background:**

Linoleic acid is an important polyunsaturated fatty acid, required for all eukaryotes. Microsomal delta-12 (Δ^12^) oleate desaturase (FAD2) is a key enzyme for linoleic acid biosynthesis. Desert shrub *Artemisia sphaerocephala* is rich in linoleic acid, it has a large *FAD2* gene family with twenty-six members. The aim of this work is to unveil the difference and potentially functionality of *AsFAD2* family members.

**Results:**

Full-length cDNAs of twenty-one *AsFAD2* genes were obtained from *A. sphaerocephala*. The putative polypeptides encoded by *AsFAD2* family genes showed a high level of sequence similarity and were relatively conserved during evolution. The motif composition was also relatively conservative. Quantitative real-time PCR analysis revealed that the *AsFAD2–1* gene was strongly expressed in developing seeds, which may be closely associated with the high accumulating ability of linoleic acid in *A. sphaerocephala* seeds. Although different *AsFAD2* family members showed diverse response to salt stress, the overall mRNA levels of the *AsFAD2* family genes was stable. Transient expression of *AsFAD2* genes in the *Nicotiana benthamiana* leaves revealed that the encoded proteins were all located in the endoplasmic reticulum. Heterologous expression in *Saccharomyces cerevisiae* suggested that only three *As*FAD2 enzymes, *As*FAD2–1, − 10, and − 23, were Δ^12^ oleate desaturases, which could convert oleic acid to linoleic acid, whereas *As*FAD2–1 and *As*FAD2–10 could also produce palmitolinoleic acid.

**Conclusions:**

This research reported the cloning, expression studies, subcellular localization and functional identification of the large *AsFAD2* gene family. These results should be helpful in understanding fatty acid biosynthesis in *A. sphaerocephala*, and has the potential to be applied in the study of plant fatty acids traits.

## Background

Linoleic acid (LA, C18:2) is known as an important polyunsaturated fatty acid (PUFA), required for normal growth of all eukaryotes [1]. LA is a precursor for the synthesis of other PUFAs such as linolenic acid and arachidonic acid, and physiologically active regulatory compounds such as prostaglandin [2]. LA has the effect of lowering serum cholesterol and triglyceride levels, which is good for preventing cardiovascular diseases, such as atherosclerosis and myocardial infarction [3]. In addition, LA is also the precursor of conjugated linoleic acid (CLA), which is mainly generated in the rumen of ruminant animals and has been shown to enhance immune function and exert multiple beneficial effects in obesity, cancer, inflammatory diseases, and hypertension [4, 5]. However, LA cannot be synthesized by humans and other mammals, and must be consumed through diet to support normal physiological metabolism [6]. In plants, higher LA content helps maintaining the fluidity and integrity of the cell membrane, which is beneficial for their adaptation to various biotic or abiotic stresses [7, 8].

LA synthesis in plants is usually catalyzed by membrane-bound enzymes Δ^12^ fatty acid desaturases (FADs), it is also known as ω-6 FADs, which act by introducing a double bond at the delta-12 (Δ^12^) position of the oleic acid carbon chain [9]. According to the location in the endoplasmic reticulum (ER) or plastids, Δ^12^ FADs are divided into microsomal (FAD2) and plastid (FAD6) enzymes. In recent years, *FAD2* genes have been identified and functionally analyzed in a variety of organisms, including plants, fungi, and some other lower animals [1, 10]. To date, the *FAD2* gene has been cloned from many plant species. With the exception of *Arabidopsis thaliana*, which has only one *FAD2* gene [11], most plants have multiple *FAD2* genes. Thus, olive (*Olea europaea*) has two *FAD2* genes [12], Oilseed rape (*Brassica napus*) has four [13], peanut (*Arachis hypogaea*) has six [14], safflower (*Carthamus tinctorius*) has eleven [15], and *Artemisia sphaerocephala* has twenty-six [16]. Gene families usually occur through gene duplication and mutations, and the variations in the number of gene family members is an important evolutionary mechanism underlying functional diversity and shaping genomic adaptation in various species [17]. Therefore, different numbers of the *FAD2* genes in plants may be a result of their adaptation to diverse environmental conditions.

The FAD2 enzymes play an important role in plant fatty acid synthesis, and consequently, in their growth, development, and resistance to low temperatures and high salt concentrations, as well as other biotic and abiotic stresses [8]. Thus, it was found that *Arabidopsis* lacking the *FAD2* gene has reduced tolerance to cold [18] and increased sensitivity to salt at the seed germination and seedling stages [19]. The safflower *CtFAD2–1* gene, which was expressed in developing seeds, was mostly responsible for the desaturation of storage lipids; thus, *CtFAD2–3, − 4, − 6,* and *− 7* were mainly expressed in the cotyledons and hypocotyls of seedlings, whereas *CtFAD2–5* and *− 8* were specifically expressed in roots and *CtFAD2–10* in flowers, were mostly responsible for the desaturation of membrane lipids [15]. In cotton, the expression of *FAD2–3* and *FAD2–4* genes were induced under cold stress, whereas that of *FAD2–2* was not affected [20]. Heterologous expression of sunflower *FAD2–1* and *FAD2–3* genes in yeast cells resulted in the increase of dienoic fatty acid content, which give the help of enhancing the freeze and salt tolerance of yeast [21]. Two *ShFAD2* genes from *Salvia hispanica* shared a similar expression pattern, either induction or suppression, in response to various abiotic stresses [22]. Overall, these findings indicated that different *FAD2* genes of the same plant may vary not only in their tissue expression patterns and functional characteristics, but also in responses to environmental stresses. At present, the research on *FAD2* genes is mainly conducted in model plants and oil crops, and there is no information on the expression and functional activity of *FAD2* genes from the desert plant *A. sphaerocephala*, which has the largest *FAD2* gene family among the investigated plants.

*Artemisia sphaerocephala* Kraschen, which belongs to the *Artemisia* genus of the *Compositae* family, is a perennial wild shrub widely distributed in the moving and semi-stable sand dunes in the deserts of northern China [23]. *Artemisia sphaerocephala* seeds contain 21.5% oil and can be used to produce biodiesel [24], nearly 90% of seeds oil are unsaturated fatty acids, especially LA, constituting over 78% of total fatty acids [25]. Comparing with other plants, such as sunflower, soybean, and peanut, *A. sphaerocephala* seeds and leaves can accumulate much more LA [16]. The maintenance of high degree of membrane lipid unsaturation under stress conditions is one of the important stress adaptation mechanisms in plants, previous studies showed that *A. sphaerocephala* is resistant to drought and salt by maintaining high LA content [26, 27]. Twenty-six *FAD2* genes were identified in *A. sphaerocephala*, which is the largest *FAD2* gene family reported till now [16]. In this study, we cloned full-length cDNA of the *A. sphaerocephala FAD2* (*AsFAD2*) gene family members, and analyzed their structural characteristics, tissue distribution, and expression levels under high salt stress conditions. Using heterologous expression systems, we also evaluated subcellular localization and functional activity of *As*FAD2 proteins. These results should be helpful in further understanding of the roles of the *AsFAD2* gene family in the maintenance of high LA content in *A. sphaerocephala*.

## Results

Cloning and analysis of the full-length cDNA of *AsFAD2* gene family.

We cloned the full-length cDNAs of twenty-one *AsFAD2* genes from different *A. sphaerocephala* tissues using reverse transcription PCR (RT-PCR) and rapid amplification of cDNA ends (RACE) methods based on transcriptome sequence data (Additional file 1: Table S1). However, because of the short lengths of the core fragments and low expression levels of these genes in the tissue the full-length cDNAs of *AsFAD2–3, − 17, − 18, − 25,* and *− 26* genes were not obtained. The size of full-length cDNAs for the twenty-one *AsFAD2* genes varied between 1320 and 1728 bp, whereas the length of 5′ UTRs and 3′ UTRs were between 27 and 373 bp and 87–279 bp, respectively, and the predicted protein sizes were between 371 and 429 amino acids. The theoretical molecular masses and isoelectric points of predicted proteins were about 43.50–49.13 and 6.22–8.83, respectively. According to grand average of hydropathicity (GRAVY) analysis, *AsFAD2–2, − 7, − 14,* and *− 23* genes encoded hydrophobic proteins, whereas the other genes encoded hydrophilic proteins, as they had positive and negative GRAVY values differently. The predicted transmembrane number was between 3 and 6. Plant-mPLoc analysis predicted that the twenty-one *AsFAD2* genes were located in the ER.

Sequence identity of multiple members of *AsFAD2* gene family.

Sequence similarity among the coding regions of twenty-one *AsFAD2* genes at the amino acid level was presented in Additional file 2: Fig. S1. The result showed that the pairwise similarity of *As*FAD2–1 and *As*FAD2–12, *As*FAD2–16 and *As*FAD2–19 were identical with the similarity level of 100.00%, whereas there was only one amino acid different in the pairwise similarity of AsFAD2–5 and *As*FAD2–16/19, *As*FAD2–6 and *As*FAD2–24, *As*FAD2–7 and *As*FAD2–14, namely, the similarity levels among these amino acid sequences were 99.74%. Thus, the *As*FAD2–1, *As*FAD2–5, *As*FAD2–6, and *As*FAD2–7 were selected for further study. The putative amino acid sequences of sixteen *AsFAD2* genes were significantly different, and the similarity level range from 36.54 to 97.85%.

Phylogenetic and motif analysis of encoded proteins of *AsFAD2* gene family.

**Fig. 1:**
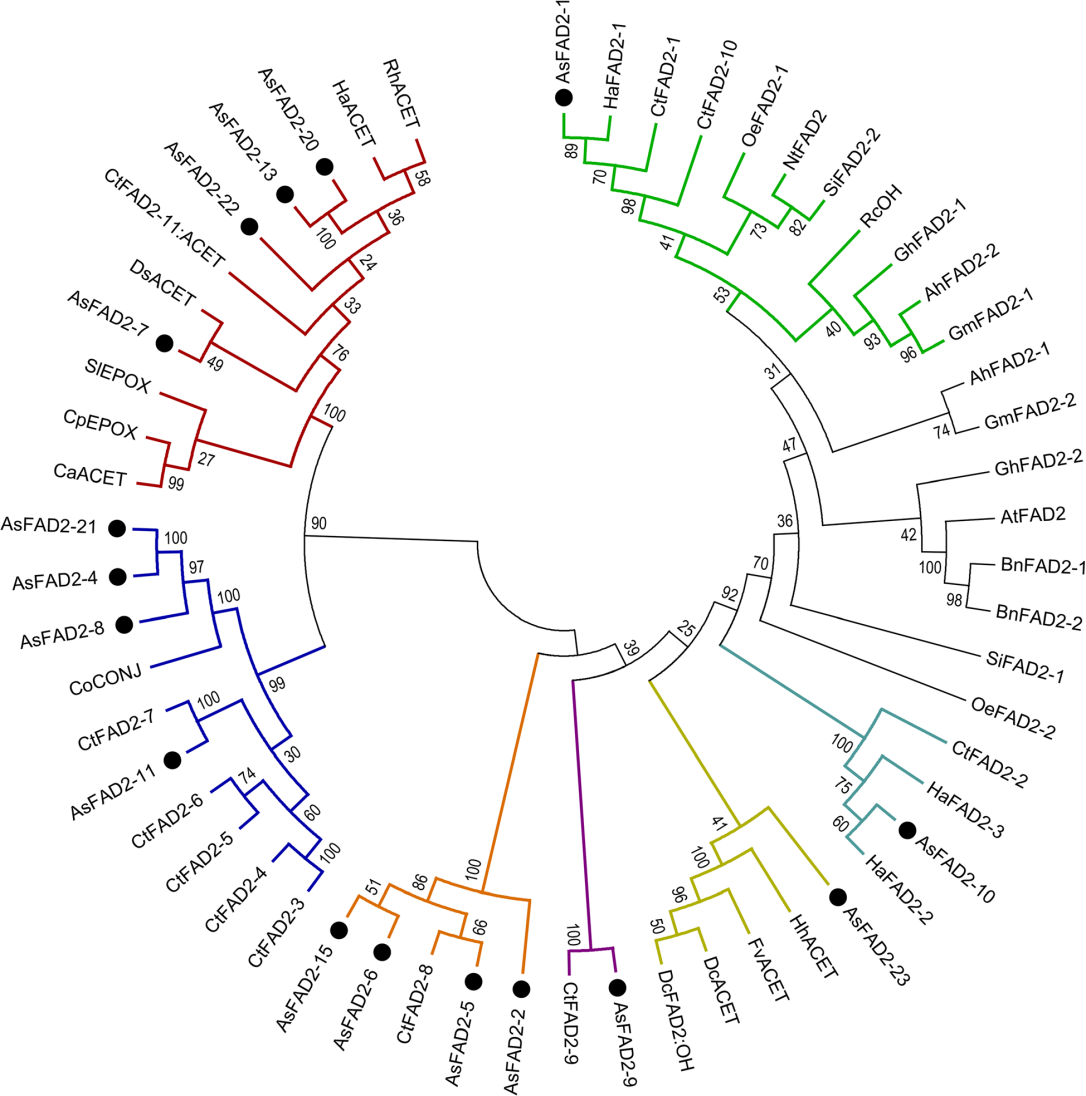
Phylogenetic comparison of *As*FAD2s and other plants FAD2s*.* The phylogenetic tree was generated by MEGA6.0. FAD2 desaturases (FAD), hydroxylases (OH), epoxygenases (EPOX), acetylenases (ACET) and conjugases (CONJ) from various plants were included in the alignment. The GenBank accession numbers of the amino acid sequences represented in the phylogenetic tree were: *Ah*FAD2–1, ACZ06072.1; *Ah*FAD2–2, AHN60569.1; sixteen *As*FAD2 proteins; *At*FAD, AAM61113.1; *Bn*FAD2–1, AAF78778.1; *Bn*FAD2–2, AAS92240.1; *Ca*ACET, ABC00769.1; *Co*CONJ, AAK26632.1; *Cp*EPOX, CAA76156.1; *Ct*FAD2–1, AGC65498.1; *Ct*FAD2–2, AGC65499.1; *Ct*FAD2–3, AGC65500.1; *Ct*FAD2–4, AGC65501.1; *Ct*FAD2–5, AGC65502.1; *Ct*FAD2–6, AGC65503.1; *Ct*FAD2–7, AGC65504.1; *Ct*FAD2–8, AGC65505.1; *Ct*FAD2–9, AGC65506.1; *Ct*FAD2–10, AGC65507.1; *Ct*FAD2–11:ACET, AGC65508.1; *Dc*ACET, AAO38033.1; *Dc*FAD2:OH, AAK30206.1; *Ds*ACET, AAO38036.1; *Fv*ACET, AAO38034.1; *Gh*FAD2–1, CAA65744.1; *Gh*FAD2–2, CAA71199.1; *Gm*FAD2–1, AAB00859.1; *Gm*FAD2–2, AAB00860.1; *Ha*FAD2–1, AAL68981.1; *Ha*FAD2–2, AAL68982.1; *Ha*FAD2–3, AAL68983.1; *Ha*ACET, ABC59684.1; *Hh*ACET, AAO38031.1; *Nt*FAD, AAT72296.2; *Oe*FAD2–1, AAW63040.1; *Oe*FAD2–2, AAW63041.1; *Rc*OH, AAC49010.1; *Rh*ACET, AAO38035.1; *Si*FAD2–1, XP_011075145.1; *Si*FAD2–2, XP_011080227.1; and *Sl*EPOX, AAR23815.1 (*Ah*, *Arachis hypogaea*; *As*, *Artemisia sphaerocephala*; *At*, *Arabidopsis thaliana*; *Bn*, *Brassica napus*; *Ca*, *Crepis alpine*; *Co*, *Calendula officinalis*; *Cp*, *Crepis palaestina*; *Ct*, *Carthamus tinctorius*; *Dc*, *Daucus carota*; *Ds*, *Dimorphotheca sinuate*; *Fv*, *Foeniculum vulgare*; *Gh*, *Gossypium hirsutum*; *Gm*, *Glycine max*; *Ha*, *Helianthus annuus*; *Hh*, *Hedera helix*; *Nt*, *Nicotiana tabacum*; *Oe*, *Oleaeuropaea*; *Rc*, *Ricinus communis*; *Rh*, *Rudbeckia hirta*; *Si*, *Sesamum indicum*; *Sl*, *Stokesia laevis*)

To elucidate phylogenetic relationship of the *AsFAD2* gene family, the deduced polypeptide sequences of the selected sixteen *AsFAD2* genes (Additional file 3: Table S2) were aligned with FAD2 sequences of other plants, including oil plants, model plants, and some plant with divergent FAD2 fatty acid modifying enzymes (Fig. 1). Phylogenetic analysis showed that the sixteen *As*FAD2 were divided into seven groups. *As*FAD2–1 was clustered with other seed expressed FAD2s, such as sunflower *Ha*FAD2–1 and safflower *Ct*FAD2–1. *As*FAD2–10 was clustered together with other constitutively expressed FAD2s, such as sunflower *Ha*FAD2–2, *Ha*FAD2–3, and safflower *Ct*FAD2–2. *As*FAD2–23 was clustered together with fatty acid acetylenases and hydroxylases from other plants. *As*FAD2–9 and *Ct*FAD2–9, *As*FAD2–2, − 5, − 6, − 15 and *Ct*FAD2–8, and *As*FAD2–11 and *Ct*FAD2–7 were positioned next to each other, respectively, in the same branch. *As*FAD2–4, − 8, and − 21 were clustered with fatty acid conjugases from *Calendula officinalis*. *As*FAD2–7, − 13, − 20, and − 22 proteins were clustered together with fatty acid acetylenases and epoxygenases from several plant species.

**Fig. 2:**
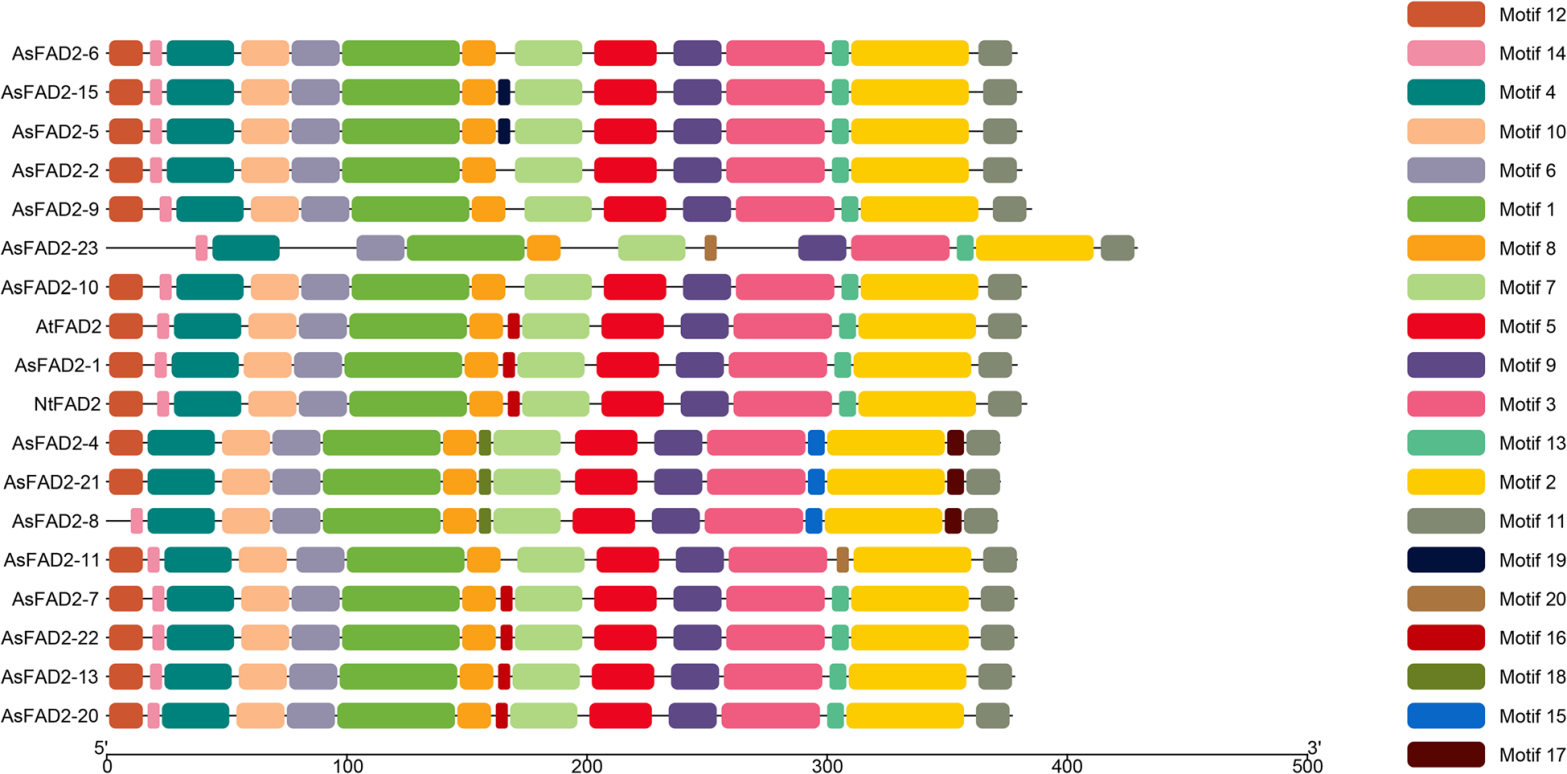
Phylogenetic relationship and conserved motif composition of *As*FAD2 family proteins. Applied parameters of MEME were as following: motif numbers, twenty; minimum width for each motif, six; maximum width for each motif, fifty. Different motifs were indicated by different colors

The alignment of putative *As*FAD2 polypeptides together with selected plant orthologs was shown in Additional file 4: Fig. S2. The *As*FAD2 polypeptides contained C-terminal aromatic amino acid-rich motifs. For example, *As*FAD2–1, *As*FAD2–2, and *As*FAD2–4 had YKNKM, FKNKL and WFKK, respectively. Additionally, *As*FAD2 family proteins contained three highly conserved histidine-rich motifs. Motifs of FAD2 protein sequences of sixteen *A. sphaerocephala*, one *Arabidopsis thaliana* and one *Nicotiana tabacum* were analyzed (Fig. 2). The detailed information of twenty putative conserved motifs were shown in Additional file 5: Fig. S3. These proteins all had nine conserved motifs, including motif 1, 2, 3, 4, 6, 7, 8, 9, and 11. The motif composition of the *As*FAD2 family proteins was relatively conserved. *As*FAD2–2, − 5, − 6 and − 15 were clustered together to be a branch (Fig.1), and they all had fourteen identical motifs, *As*FAD2–5 and *As*FAD2–15 contained motif 19. *As*FAD2–9 and *As*FAD2–2 were next to each other, and had same motifs. The motif composition of *As*FAD2–23 was different from other *As*FAD2*s*. *As*FAD2–10, *At*FAD2, *As*FAD2–1 and *Nt*FAD2 were clustered together to be a branch (Fig. 1). The motifs of *At*FAD2, *As*FAD2–1 and *Nt*FAD2 were completely identical. *As*FAD2–10 lacked motif 16. *As*FAD2–4, − 8, − 11 and − 21 were located next to each other and formed a branch (Fig. 1), *As*FAD2–4 and *As*FAD2–21 had same motifs. In contrast, *As*FAD2–8 contained motif 14 and lacked motif 12, *As*FAD2–11 had motif 12. *As*FAD2–7, − 13, − 20 and − 22 were situated next to each other and formed a branch (Fig. 1), and they all had fifteen same motifs.

Expression analysis of *AsFAD2* gene family in *A. sphaerocephala.*

**Fig. 3:**
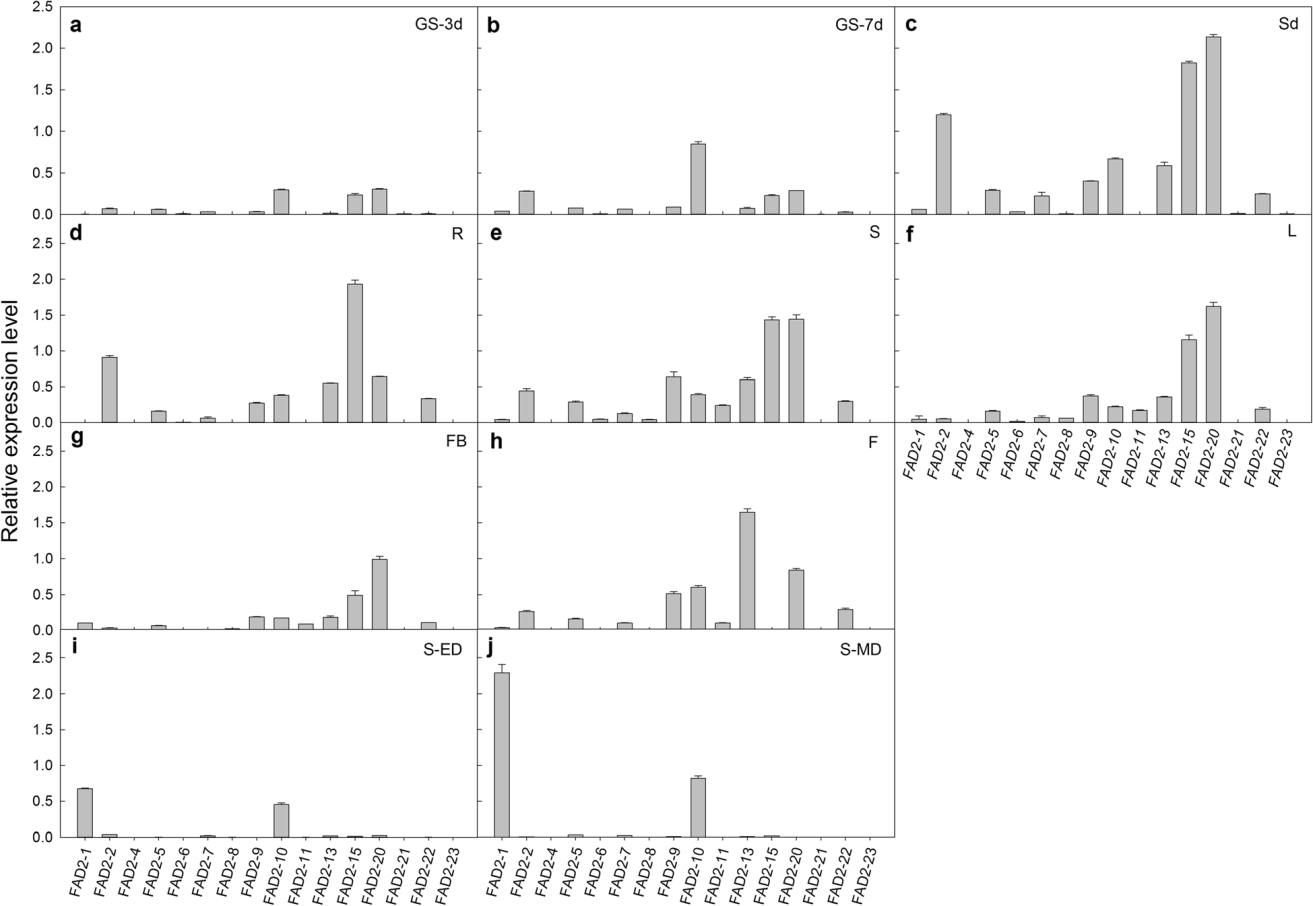
Relative expression levels of sixteen *AsFAD2* genes in various *A. sphaerocephala* tissues. **a** GS-3d (Germinated Seeds after 3 days). **b** GS-7d (Germinated Seeds after 7 days). **c** Sd (Seedlings). **d** R (Roots). **e** S (Stems). **f** L(Leaves). **g** FB (Flower Buds), **h** F (Flowers). **i** S-ED (Early Developing Seeds). **j** S-MD (Mid-Developing Seeds). The *actin* gene was used as an internal reference gene. The graph shows average values of three replicates with the respective error bars indicative of standard error

The transcript levels of the sixteen *AsFAD2* genes in different *A. sphaerocephala* tissues were detected using quantitative real-time PCR (qRT-PCR). It displayed that the expression patterns of *AsFAD2* gene family were diverse, and they may play different functions roles in different tissues and organs. The numbers and relative expression levels of *AsFAD2* genes increased significantly in the seed swelling and germination, especially the expression levels of *AsFAD2–2, − 15, − 20* increased significantly (Fig. 3a-c). The expression level of *AsFAD2–15* was the highest in roots (Fig. 3d). *AsFAD2–15* and *AsFAD2–20* showed high expression levels in stems and leaves (Fig. 3d-f). In flower buds and flowers, the expression levels of *AsFAD2–20* and *AsFAD2–13* were the highest (Fig. 3g-h), respectively, compared to other *AsFAD2s*. *AsFAD2–1* was strongly expressed in developing seeds, but had low expression levels in other tissues, belonging to gene of the seed-type expression. *AsFAD2–10* was expressed in all the checked tissues, belonging to gene of the constitutive expression. *AsFAD2–1* and *AsFAD2–10* may play an important role in the formation of high linoleic acid in *A. sphaerocephala* seeds (Fig. 3i-j).

**Fig. 4:**
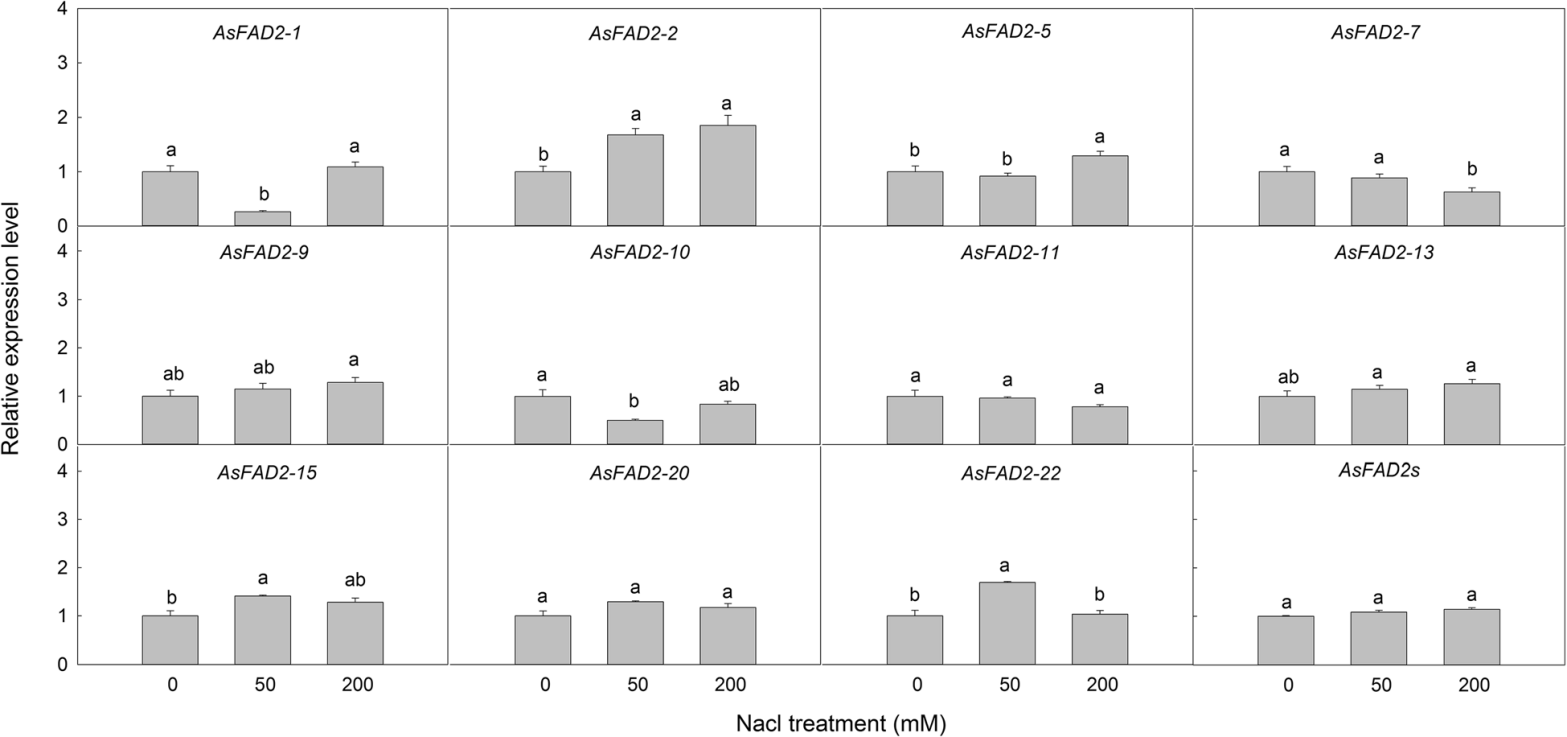
Expression analysis of eleven *AsFAD2* genes in *A. sphaerocephala* leaves treated with NaCl. Values are means ± SE (*n* = 5) and bars indicate SE. Different letters within a column indicated significant difference at *P* < 0.05

According to the expression pattern of *AsFAD2* genes in various *A. sphaerocephala* organs, eleven genes with high expression in leaves were selected to analyze their response to salt stress (Fig. 4). Forty-five day-old seedlings were treated with 50 and 200 mM NaCl for 7 days, and the relative expression of the *AsFAD2* genes in leaves was compared with that in untreated control plants. At 50 mM NaCl, the expression of *AsFAD2–1* and *− 10* were downregulated significantly, whereas that of *AsFAD2–2, − 15,* and *− 22* were upregulated, and that of the other genes were unchanged compared to control. At 200 mM NaCl, the expression of *AsFAD2–2* and *− 5* genes were increased significantly, whereas that of *AsFAD2–7* was decreased significantly, and that of the other genes showed no difference compared to control. Overall, mRNA expression of eleven *AsFAD2* genes was not significantly changed with NaCl treatment.

Subcellular Localization of *As*FAD2 proteins.

**Fig. 5:**
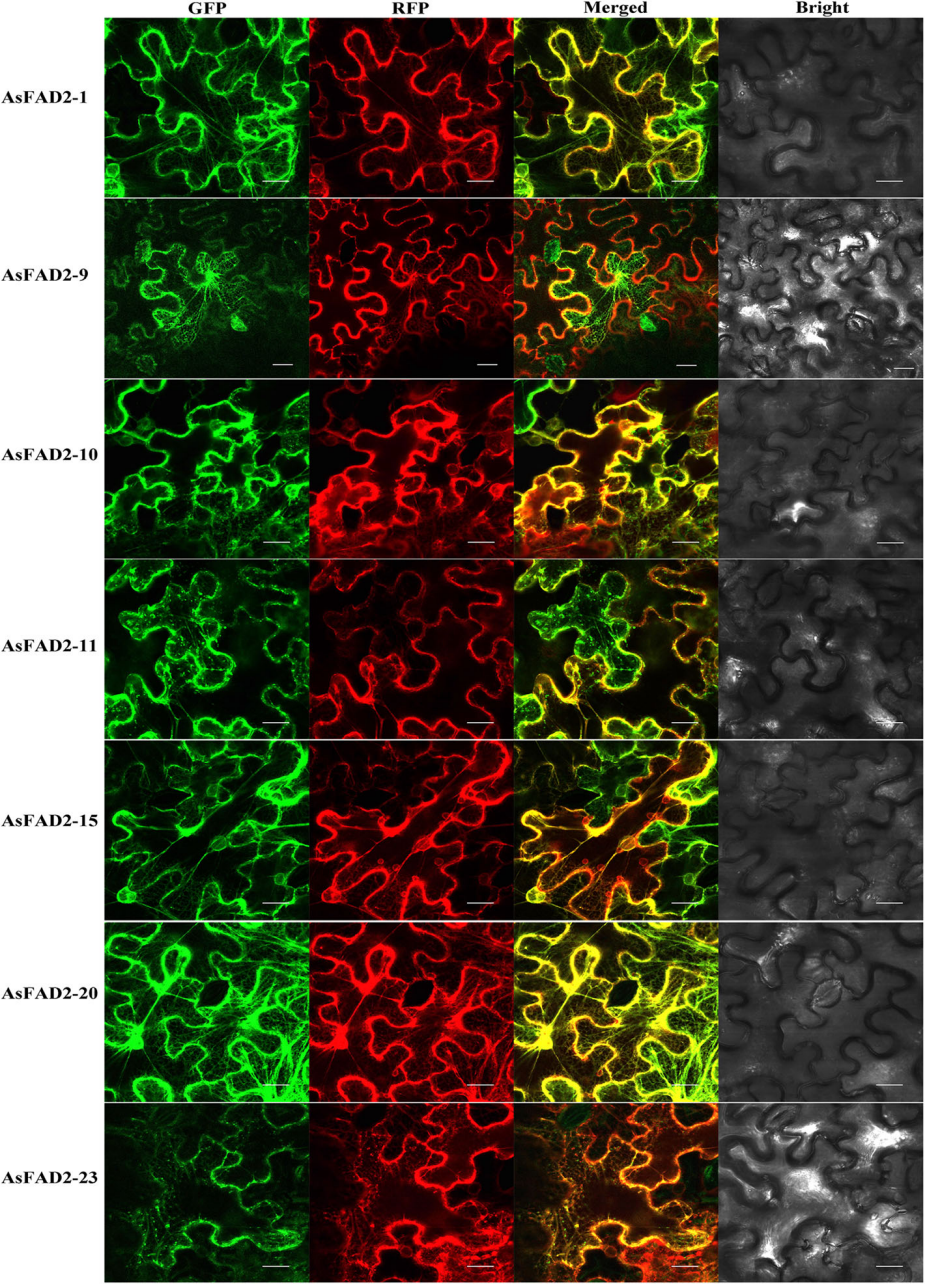
Subcellular localization of seven *As*FAD2s. Confocal laser scanning microscopy images of *N*. *benthamiana* leaf cells expressing *As*FAD2 proteins with GFP fused to their C-termini (*As*FAD2-GFP). HDEL-RFP (ER-RK) was used as an indicator of the ER. Scale bars =20 μm

Based on phylogenetic relationship and tissue expression patterns, seven *AsFAD2* genes were selected for subcellular localization analysis, including *AsFAD2–1, − 9, − 10, − 11, − 15, − 20,* and *− 23*. The results showed that seven *As*FAD2 cDNA-encoded proteins were localized to network-like organelles, the strong green fluorescent protein (GFP) and red fluorescent protein (RFP) signals were observed in the epidermal cells of tobacco leaves, and the both fluorescent signals could be overlapped and displayed as yellow fluorescent signals, indicating that the selected seven *As*FAD2s were transiently expressed in the ER of tobacco leaf epidermal cells (Fig. 5). It was speculated that the other *As*FAD2 proteins could also be located in the ER.

Functional analysis of *AsFAD2* genes in yeast.

**Fig. 6:**
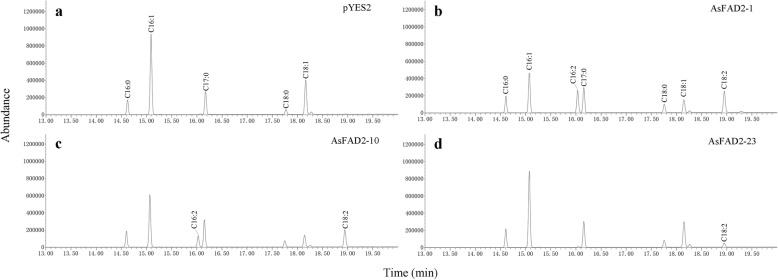
GC-MS of yeast cultures transformed with the pYES2 vector alone (**a**), pYES2 with *As*FAD2–1 (**b**), pYES2 with *As*FAD2–10 (**c**), pYES2 with *As*FAD2–23 (**d**). C16:0, palmitic acid; C16:1, palmitoleic acid; C16:2, palmitolinoleic acid; C18:0, stearic acid; C18:1, oleic acid; C18:2, linoleic acid; C17:0 as internal standard

Sixteen *AsFAD2* family members were expressed in the *Saccharomyces cerevisiae* INVSc1, and the fatty acid compositions of the yeasts were analyzed (Fig. 6 and Additional file 6: Table S3). The results indicated that dienoic fatty acids, including palmitolinoleic acid (C16:2) and LA (C18:2), were not produced in the yeast with the empty pYES2 vector (Fig. 6a). However, C18:2 content was respectively 18.58, 16.54 and 3.29% of total fatty acids in the transformed yeast expressing the *AsFAD2–1* (Fig. 6b), *AsFAD2–10* (Fig. 6c), and *AsFAD2–23* (Fig. 6d), the conversion ratio of C18:1 to C18:2 were 60.07, 57.49 and 12.78%, respectively (Additional file 6: Table S3). In addition, C16:2 was detected in the transformed yeast strains expressing *AsFAD2–1* and *AsFAD2–10*, C16:2 content was respectively 18.10 and 9.95%, and the conversion ratio were 36.41 and 18.82%, respectively (Additional file 6: Table S3). However, no corresponding fatty acid product was detected in yeast cells expressing other genes (Additional file 6: Table S3).

## Discussion

Among plant species examined to date, *A. sphaerocephala* had the largest *FAD2* family containing twenty-six genes, which was much more than in the next largest family in safflower (11 genes) [15]. In this study, we isolated twenty-one *AsFAD2* genes from *A. sphaerocephala* (Additional file 1: Table S1), including sixteen *AsFAD2* genes with distinct coding regions (Additional file 2: Fig. S1). The *AsFAD2* family members contained uninterrupted coding region sequences, which were highly homologous and relatively conserved during evolution (Fig. 2). Similarly, in safflower, the coding regions of *CtFAD2* genes did not contain introns. Therefore, the formation of the gene family was suggested to be most likely caused by gene duplication rather than nucleotide alternative splicing [15]. Whole-genome sequencing of soybean revealed two genome duplication events occurred fifty-nine and thirteen million years ago [28], and seven soybean *FAD2* genes were generated as a result in previous study [29]. In cucumber, two *FAD2* genes also originated through gene duplication [30]. *A. sphaerocephala* is a cross-pollinated diploid wild plant [31], in the absence of genomic data, it is unclear whether this species has undergone whole-genome duplication or not. Therefore, further research was needed to determine how *FAD2* gene family was emerged in *A. sphaerocephala* with a large quantity. However, based on the results of this study, it could be inferred that the formation of *AsFAD2* gene family may be similar to that in safflower, i.e., could be a result of gene duplication.

The deduced amino acid sequences of the *AsFAD2* family members contain aromatic amino acid-rich motifs at the C-terminus and three highly conserved histidine-rich motifs, which had similarities as well as differences compared with those in other plants (Additional file 4: Fig. S2), indicated complexity of the *AsFAD2* gene family and more possibilities for the diversification of *As*FAD2 enzymes. In addition, the predicted *As*FAD2 proteins contain between three and six transmembrane regions (Additional file 1: Table S1), which played an important role in FAD2 catalytic activity [32], and confirmed that the *A. sphaerocephala* FADs were membrane-bound. The number of transmembrane domains among plant FAD2 enzymes is different, it is usually in the range of three to six. Thus, red flax (*Linum grandiflorum*), pumpkin (*Cucurbita pepo*), sesame (*S. indicum*), and grape (*Vitis labrusca*) enzymes contain 3, 4, 5, and 6 transmembrane regions, respectively [33, 34]. Our data indicated that the *AsFAD2* family members were structurally diverse.

The FAD2 enzymes not only have desaturase activity, but can also perform other fatty acid modifications, including hydroxylation [35], epoxidation [36], and formation of acetylene bonds [37, 38] and conjugated double bonds [39, 40]. Some FAD2 enzymes had more than two functions. For example, *Lf*FAD2 of *Lesquerella fendleri* was a bifunctional enzyme with dehydrogenase and hydroxylase activity [41],whereas *Crepis alpina Ca*FAD2 and safflower *Ct*FAD2–11 were tri-functional enzymes as they can introduce a carbon double bond in either cis or trans configuration or acetylenic bond at the Δ12 position [15, 42]. Based on phylogenetic relationship inferred *As*FAD2–4, − 8, − 11, and − 21 proteins may be conjugated enzymes, *As*FAD2–7, − 13, − 20, and − 22 may have acetylene and cyclooxygenase activities, and *As*FAD2–23 may have acetylase and hydroxylase activities (Fig. 1). However, no corresponding fatty acid products were detected in transgenic yeast cells (data not presented). These results illustrated that these *As*FAD2s did not have the functions of fatty acid modifying enzymes in transgenic yeast, which was consistent with the result of safflower *CtFAD2* gene family [15].

Current studies have shown that although the expression and function of *FAD2* genes in plants have temporal and spatial differences, roughly two types of expression patterns, constitutive and seed-specific, can be distinguished [43]. Thus, among five *FAD2* copies identified in soybean, *FAD2–1A* and *FAD2–1B* were expressed specifically in immature seeds, encoding enzymes responsible for the synthesis of seed polyunsaturated fatty acids, whereas *FAD2–2A, FAD2–2B*, and *FAD2–2C* were constitutive expression and encoding enzymes responsible for membrane lipid desaturation [44]. The phylogenetic analysis showed that the *AsFAD2–1* gene belonged to seed-specific expression (Fig. 1), which was consistent with its tissue expression profile (Fig. 3), whereas *AsFAD2–1* was strongly expressed in developing seeds, which was similar to the expression patterns of *FAD2–1* genes in most plants such as cotton and grape [33, 45]. The phylogenetic analysis also revealed that the *AsFAD2–10* gene was constitutively expressed (Fig. 1), which was consistent with the results of tissue expression (Fig. 3). The *AsFAD2* genes had the highest homology with the safflower *CtFAD2* genes (Fig. 1), apparently because both species belong to the same *Compositae* family, i.e., have close genetic relationship.

The regulation of *FAD2* gene is important in understanding the composition of fatty acids and biosynthesis, plant development, and essential role in biotic and abiotic stresses [8]. Under salt stress, FAD2 enzymes play a key role in regulating and maintaining lipid composition, biophysical properties, and normal function of membrane-bound proteins [8]. In high salt-exposed *Arabidopsis*, the expression of *FAD2* mutants resulted in low levels of PUFAs, which decreased membrane lipid fluidity and salt tolerance [19]. Two *ShFAD2* genes from *Salvia hispanica* were differentially upregulated or repressed by salt stress [22]. In this study, except *AsFAD2–1* and *− 10* were downregulated, *AsFAD2–2*, *− 15*, and *− 22* were upregulated at 50 mM NaCl; *AsFAD2–2* and *− 5* were increased and *AsFAD2–7* was decreased significantly at 200 mM NaCl compared to control. Other genes showed no difference compared to control under salt stress. The total expression level of eleven *AsFAD2* genes was not affected (Fig. 4). Our previous study has shown that *A. sphaerocephala* could maintain its membrane unsaturation degree at a relatively stable level under salt stress [27]. In this research, the completely opposite response of the different member of *AsFAD2* family indicates that *AsFAD2* family could help the plant to maintain the balance of oleic acid and linoleic acid under salt stress, which has not been reported before. Overall, these results suggested that the *AsFAD2* gene family members might adjust to appropriate levels to protect the cell membrane of *A. sphaerocephala* from salt stress. The relationship between the responses of these *AsFAD2* genes to various stresses and fatty acid composition of the plant need further study.

Previous study suggested that fatty acid modifications, including elongation and desaturation, occur on the ER membrane [46]. Seven *As*FAD2 proteins were located in the ER (Fig. 5), which was in agreement with previous findings for cotton FAD2–4:GFP [47], three *Bn*FAD2s:YFP [13] and *Fr*FAD2–1:GFP [48]. The above results were also consistent with the prediction of subcellular localization by Plant-mPLoc 2.0 (Additional file 1: Table S1). We deduced the other *As*FAD2 enzymes may be also ER-localized. These results further confirmed that the core reaction of LA biosynthesis in plants occurs in the ER. In addition, the fatty acid composition of *N. benthamiana* leaves expressing seven *As*FAD2s were detected, and no additional novel fatty acids was found (such as crepenynic acid) compared to the controls (data not presented). This result was different from the *Ct*FAD2–11 [15].

*S. cerevisiae* INVSc1 is a suitable heterologous expression system for functional studies of FAD2 enzymes, because it has a simple fatty acid profile, contains the FAD2 substrate (oleic acid), and lacks endogenous FAD2 activity. Functional analysis of FAD2 enzymes from many plants such as *A. thaliana* [49], Tung [50], Soybean [45], and *Camelina sativa* [51] were successfully performed in yeast, where recombinant enzymes produced certain amounts of LA. In this study, sixteen *AsFAD2* genes were expressed in *S. cerevisiae* INVSc1, which were then analyzed for fatty acid composition. It was found that *As*FAD2–1, − 10, 23 could effect the conversion of C18:1 to C18:2 in transgenic yeast, whereas no C18:2 was detected in the controls. Furthermore, *As*FAD2–1 and *As*FAD2–10 could also convert C16:1 to C16:2. These results indicated that *As*FAD2–1 and *As*FAD2–10 were both Δ^12^ oleate desaturases and Δ^12^ palmitoleate desaturase. Previous study showed that C16:2 was generated in the plastid of plant by FAD6 activity [52]. This study showed that C16:2 could also be produced by FAD2 in the ER. Similar results have been found in other studies [13, 15, 48, 51], and the reasons need further study. In this study, the corresponding fatty acid products were not detected in yeast cells expressing other *AsFAD2* genes. Similar results were obtained in safflower, where five *Ct*FAD2 family members were found to be functional and six non-functional [15]. Although the heterologous expression system in yeast is normally used to study the function of plant PUFA biosynthesis enzymes, numerous factors still mediate the enzyme activity, such as yeast strain, promoter type, and culture condition [1]. In addition, we speculated neofunctionization, pseudogenization could also cause those genes had no functions in yeast, although they could expressed in the tissues of *A. sphaerocephala*.

## Conclusions

In this study, we cloned and characterized a large *FAD2* gene family from *A. sphaerocephala*. The coding region sequences of *AsFAD2* gene family were highly homologous and relatively conservative during evolution. The expression of *AsFAD2* gene family members had temporal and spatial differences. However, the overall expression of *AsFAD2* genes remained stable under salt stress. *As*FAD2 proteins were all located in the ER. Three *As*FAD2 enzymes were confirmed in transgenic yeasts as Δ^12^ fatty acid desaturases.

## Methods

### Plant material

We used seventeen samples of *A. sphaerocephala*, including seeds after 3 and 7 days of germination, seedlings, roots, stems, leaves, flower buds, flowers, early developing, mid-developing, and mature seeds, and six different callus tissues. Leaves, stems, roots, flowers, flower buds, early developing seeds, mid-developing seeds and mature seeds were collected from *A. sphaerocephala* plants (voucher No. 0019079, identified by Quanru Liu and deposited at Hebei Normal University, http://www.nsii.org.cn/node/79/cvh/157/2ef/15103591) growing in the Alxa Desert of Inner Mongolia, northwest China (N: 38°68′, E: 105°61′). No specific permission was required for use of these materials for experimental purposes. In addition, seeds after 3 and 7 days of germination, seedlings, and six different callus tissues were collected from the laboratory of Lanzhou University, Lanzhou, China. The collection of all samples completely complies with local and national legislation permission. These samples were taken as same as our previous work [16]. One month old *Nicotiana tabacum* plants were used for transient expression of *AsFAD2* genes to determine the subcellular localization of the encoded FAD2 proteins. *N. tabacum* seeds were preserved in our lab.

Isolation of the full-length cDNA of *AsFAD2* gene family.

Total RNA was extracted from each plant sample using the UNlQ-10 Column Trizol Total RNA Isolation Kit (Sangon, China) and analyzed for concentration and quality using NanoDrop ND1000 (Thermo Fisher Scientific, USA) and gel electrophoresis. Based on our previous study [16], nucleotide sequences of twenty-six *AsFAD2* genes determined by RNA-seq (Additional file 7: Table S4). 5′/3′ RACE gene-specific primers for each *AsFAD2* gene were designed by Primer 5.0 and synthesized by the Sangon Company (Additional file 8: Table S5 and Additional file 9: Table S6). Total RNA of seventeen *A. sphaerocephala* samples (1 μg for each) was used as a template to synthesize first-strand cDNA by 5′ and 3′ RACE, respectively, using a SMARTer® RACE 5′/3′ Kit (Clontech, Japan) according to the manufacturer’s instructions. Finally, the full-length cDNA of each *AsFAD2* gene was obtained by splicing of 5′ and 3′ sequences and reference sequences using the DNAMAN 6.0 software.

The open reading frame (ORF) of each *AsFAD2* gene was identified using the online ORF finder software (https://www.ncbi.nlm.nih.gov/orffinder/). Primers were designed based on the region upstream of the start codon and downstream of the stop codon (both codons were included) (Additional file 10: Table S7) and ORFs were amplified by PCR using PrimeSTAR HS DNA Polymerase (Takara, Japan). The resultant products were purified by the TaKaRa MiniBEST Agarose Gel DNA Extraction Kit (Takara, Japan), subcloned into the pLB vector (Tiangen, China), and used to transform *E. coli* (Transgen, China). All constructs were verified by sequencing.

Bioinformatics analysis.

The characteristics of *AsFAD2* genes were analyzed using several online resources. Nucleotide and deduced amino acid sequences were identified by NCBI BLAST (http://www.ncbi.nlm.nih.gov/BLAST/) and physicochemical properties of putative proteins predicted using ProtParam (http://web.expasy.org/protparam/). TMHMM (http://www.cbs.dtu.dk/services/TMHMM/) and Plant-mPLoc 2.0 (http://www.csbio.sjtu.edu.cn/bioinf/plant-multi/) servers were used to predict transmembrane regions and subcellular location, respectively. Sequence motifs were searched and analyzed using the MEME web server (http://meme-suite.org/tools/meme) and TBtools software. A phylogenetic tree was constructed by MEGA6.0 using the maximum likelihood method, and bootstrapping with 1000 replicates was used to establish the confidence limit of the tree branches.

Quantitative real-time PCR (qRT-PCR) analysis.

Total RNA was extracted from ten tissues of *A. sphaerocephala* (germinated seeds after 3 and 7 days, seedlings, roots, stems, leaves, flower buds, flowers, early and mid-developing seeds) using an RNA Isolation kit (Sangon, China), reverse-transcribed into cDNA using the PrimeScript RT reagent Kit With gDNA Eraser (Takara, Japan), and analyzed by qRT-PCR in an ABI 7500 thermocycler (Applied Biosystems, USA) using a SYBR Premix Ex Taq Kit (Takara, Japan). Primers for the sixteen *AsFAD2* genes were presented in Additional file 11: Table S8. PCR conditions were as follows: 95 °C for 30 s, and 40 cycles of 95 °C for 5 s and 60 °C for 1 min. Relative gene expression was calculated by the 2^-ΔCt^ method [53] and presented as the mean ± SE of three replicates, the actin-encoding gene was used as an internal control.

Subcellular localization of *AsFAD2* genes in *Nicotiana benthamiana* leaves.

To observe the subcellular localization of *As*FAD2 proteins, the coding sequences of selected seven representative *AsFAD2* genes without the stop codons were respectively amplified by PCR using primers listed in Additional file 12: Table S9 and then inserted into the *Xho* I and *Sal* I sites of the pBI121-EGFP vector (Miaolingbio, China) using the In-Fusion® HD Cloning Kit (Takara, Japan). So, the DNA fragments of target genes were respectively fused to the N-terminal region of GFP under the control of the CaMV35S promoter. The recombinant vectors were named as p*As*FAD2:EGFP. The pHDEL:RFP (mCherry) plasmid was used to mark the ER. The p*As*FAD2:EGFP and pHDEL:RFP were independently transformed into the *Agrobacterium tumefaciens* GV3101. The two cultures (OD_600_ = 0.8) were mixed (1:1) and co-infiltrated into epidermal tissues of *N*. *benthamiana* leaves using infiltration buffer (10 mM MES, 10 mM MgCl_2_·6H_2_O, 100 μM acetosyringone, PH = 5.7) [54]. Transfected leaves regions were examined at 48 h after injection, and analyzed with a confocal laser scanning microscope (FV1000 MPE, Olympus) at the excitation wave lengths of 488 and 561 nm to visualize GFP and RFP fluorescence, respectively.

Heterogonous expression of *AsFAD2* genes in *Saccharomyces cerevisiae.*

The coding sequences of sixteen *AsFAD2* genes were amplified using specific primers (Additional file 13: Table S10) and inserted into the shuttle vector pYES2 (Invitrogen, USA), which harbored the GAL1 promoter for inducing gene expression by galactose [47]. The resultant constructs were sequenced and introduced into *Saccharomyces cerevisiae* INVSc1 (Invitrogen, USA) using a Quick & Easy Yeast Transformation Mix kit (Takara, Japan). Yeast colonies were selected on synthetic complete medium lacking uracil (SC-U) and containing 2% glucose (w/v), and single colonies were grown in liquid medium at 30 °C with shaking for 24 h. Yeast cells were harvested by centrifugation at 1500 g for 5 min, diluted to OD_600_ = 0.4, and induced using SC-U liquid medium with 2% galactose and 1% raffinose at 22 °C for 3 days. Cells were harvested by centrifugation, washed in sterile water three times, and freeze-dried in a lyophilizer.

Total fatty acids were extracted from 0.5 g of yeast cells, and fatty acid methyl esters were analyzed by gas chromatography (Agilent 6890 N, USA) and mass spectrometry (Agilent 5975C) using a polar capillary column (Agilent DB-FFAP) as previously described [55]; heptadecanoic acid (C17:0) was used as internal standard.

### Statistical analysis

Data were subjected to one-way analysis of variance (ANOVA) using SPSS 17.0 (SPSS Inc., Chicago, IL, USA). The significant differences among means were identified by Duncan’s multiple range tests at a significance level of *P* < 0.05. Data were presented as means ± SE (*n* = 3).

## Supplementary information

**Additional file 1: Table S1.** Analysis of the full-length cDNA sequences of *AsFAD2* gene family and its predicted amino acid sequence characteristics.

**Additional file 2: Figure S1.** Sequence similarity of the coding region deduced amino acids of twenty-one *AsFAD2* genes.

**Additional file 3: Table S2.** Sixteen *As*FAD2 putative polypeptide sequences from *A. sphaerocephala.*

**Additional file 4: Figure S2.** The alignment of the putative polypeptide sequences of *AsFAD2* genes together with those of selected ortholog plants.

**Additional file 5: Figure S3.** The detailed information of putative twenty conserved motifs.

**Additional file 6: Table S3.** Fatty acid composition of transgenic yeast cells.

**Additional file 7: Table S4.** Nucleotide sequences of twenty-six *FAD2* unigenes from *A. sphaerocephala* transcriptome.

**Additional file 8: Table S5.** Primers used in the 5’RACE of twenty-one *AsFAD2* genes in *A. sphaerocephala*.

**Additional file 9: Table S6.** Primers used in the 3’RACE of twenty-one *AsFAD2* genes in *A. sphaerocephala*.

**Additional file 10: Table S7.** Primers used for amplification of the ORF of sixteen *AsFAD2* genes in *A. sphaerocephala*.

**Additional file 11: Table S8.** Primers used for qRT-PCR study of sixteen *AsFAD2* genes in *A. sphaerocephala*.

**Additional file 12: Table S9.** Primers used in subcellular localization study of seven *AsFAD2* genes.

**Additional file 13: Table S10.** Primers carrying restriction endonuclease used in heterologous expression of sixteen *AsFAD2* genes in *Saccharomy cescerevisiae.*

## Data Availability

All the data pertaining to the present study have been included in the tables and figures of the manuscript, and the authors are pleased to share all the data and plant materials upon reasonable request.

## Abbreviations

AA: Amino acid
cDNA: Complementary DNA
CLA: Conjugated linoleic acid
FAD2: Microsomal delta-12 (Δ^12^) oleate desaturase
GFP: Green fluorescent protein
GRAVY: Grand average of hydropathicity
MW: Molecular weight
NCBI: National Center for Biotechnology Information
ORF: Open reading frame
PCR: Polymerase chain reaction
PI: Isoelectric point
PUFA: Polyunsaturated fatty acid
qRT-PCR: Quantitative real-time PCR
RACE: Rapid Amplification of cDNA Ends
RFP: Red Fluorescent protein
RT-PCR: Reverse transcription PCR
UTR: Untranslated region
