# Supplementary material for: Cloning and functional analysis of the FAD2 gene family from desert shrub Artemisia sphaerocephala
Source: BMC Plant Biol. 2019 Nov 8;19:481. doi: 10.1186/s12870-019-2083-5 (PMC6839233; doi:10.1186/s12870-019-2083-5)
Supplement: Supplementary file 1 — Additional file 1: Table S1. Analysis of the full-length cDNA sequences of AsFAD2 gene family and its predicted amino acid sequence characteristics. [file 12870_2019_2083_MOESM1_ESM.docx]

Table S1. Analysis of the full-length cDNA sequences of *AsFAD2* gene family and its predicted amino acid sequence characteristics.

| Name | Full-length | 5´UTR | 3´UTR | ORF | AA | MW | PI | GRAVY | TM | Location |
| --- | --- | --- | --- | --- | --- | --- | --- | --- | --- | --- |
| *AsFAD2-1* | 1478bp | 141 bp | 197bp | 1140bp | 379 | 43.81 | 8.83 | -0.046 | 5 | ER |
| *AsFAD2-2* | 1430bp | 99 bp | 185bp | 1146bp | 381 | 43.68 | 6.88 | 0.036 | 3 | ER |
| *AsFAD2-4* | 1427bp | 32 bp | 276bp | 1119bp | 372 | 43.83 | 7.35 | -0.104 | 6 | ER |
| *AsFAD2-5* | 1463bp | 119bp | 198bp | 1146bp | 381 | 44.10 | 8.43 | -0.043 | 6 | ER |
| *AsFAD2-6* | 1706bp | 352 bp | 214bp | 1140bp | 379 | 43.83 | 8.37 | -0.035 | 6 | ER |
| *AsFAD2-7* | 1408bp | 98 bp | 170bp | 1140bp | 379 | 43.89 | 8.58 | 0.028 | 3 | ER |
| *AsFAD2-8* | 1372bp | 37bp | 219bp | 1116bp | 371 | 43.50 | 8.04 | -0.076 | 5 | ER |
| *AsFAD2-9* | 1320bp | 27 bp | 135bp | 1158bp | 385 | 44.56 | 7.32 | -0.065 | 4 | ER |
| *AsFAD2-10* | 1398bp | 86 bp | 160bp | 1152bp | 383 | 43.89 | 8.10 | -0.075 | 5 | ER |
| *AsFAD2-11* | 1380 bp | 68 bp | 172bp | 1140bp | 379 | 43.86 | 6.72 | -0.120 | 5 | ER |
| *AsFAD2-12* | 1480 bp | 141 bp | 199bp | 1140bp | 379 | 43.81 | 8.83 | -0.046 | 5 | ER |
| *AsFAD2-13* | 1420 bp | 115 bp | 168bp | 1137bp | 378 | 44.09 | 8.64 | -0.036 | 4 | ER |
| *AsFAD2-14* | 1416 bp | 100 bp | 176bp | 1140bp | 379 | 43.87 | 8.58 | 0.028 | 3 | ER |
| *AsFAD2-15* | 1324 bp | 49 bp | 129bp | 1146bp | 381 | 43.96 | 8.48 | -0.054 | 6 | ER |
| *AsFAD2-16* | 1728bp | 373bp | 209bp | 1146bp | 381 | 44.09 | 8.43 | -0.041 | 6 | ER |
| *AsFAD2-19* | 1465 bp | 122bp | 197bp | 1146bp | 381 | 44.09 | 8.43 | -0.041 | 6 | ER |
| *AsFAD2-20* | 1382 bp | 56 bp | 192bp | 1134bp | 377 | 43.85 | 8.79 | -0.033 | 4 | ER |
| *AsFAD2-21* | 1540bp | 251bp | 170bp | 1119bp | 372 | 43.86 | 7.07 | -0.116 | 6 | ER |
| *AsFAD2-22* | 1472 bp | 55 bp | 277bp | 1140bp | 379 | 44.09 | 8.59 | -0.048 | 5 | ER |
| *AsFAD2-23* | 1716 bp | 147 bp | 279bp | 1290bp | 429 | 49.13 | 6.22 | 0.071 | 5 | ER |
| *AsFAD2-24* | 1342 bp | 115 bp | 87bp | 1140bp | 379 | 43.79 | 8.37 | -0.033 | 6 | ER |

Note: 5´UTR: 5´Untranslated region, 3´UTR: 3´Untranslated region (including polyA), ORF: Open reading frame, AA: Amino acid, MW: Molecular weight, kDa, PI: Isoelectric point, GRAVY: Grand average of hydropathicity, TM: the number of transmembrane domains, location: subcellular location, ER: Endoplasmic reticulum.
