# Supplementary figures and images for: Cloning and functional analysis of the FAD2 gene family from desert shrub Artemisia sphaerocephala
Source: BMC Plant Biol. 2019 Nov 8;19:481. doi: 10.1186/s12870-019-2083-5 (PMC6839233; doi:10.1186/s12870-019-2083-5)

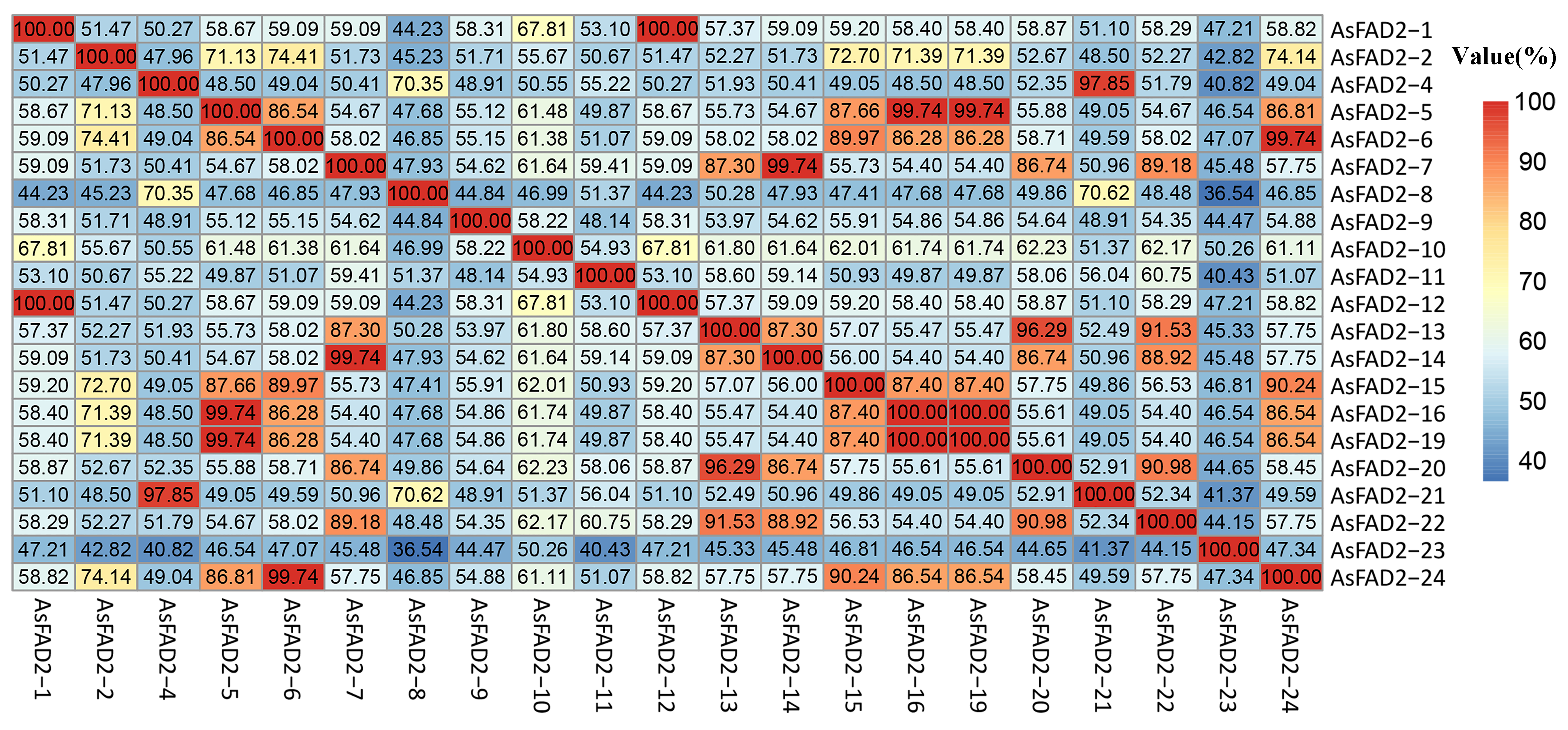

Supplement: Supplementary file 2 — Additional file 2: Figure S1. Sequence similarity of the coding region deduced amino acids of twenty-one AsFAD2 genes. [file 12870_2019_2083_MOESM2_ESM.tif]

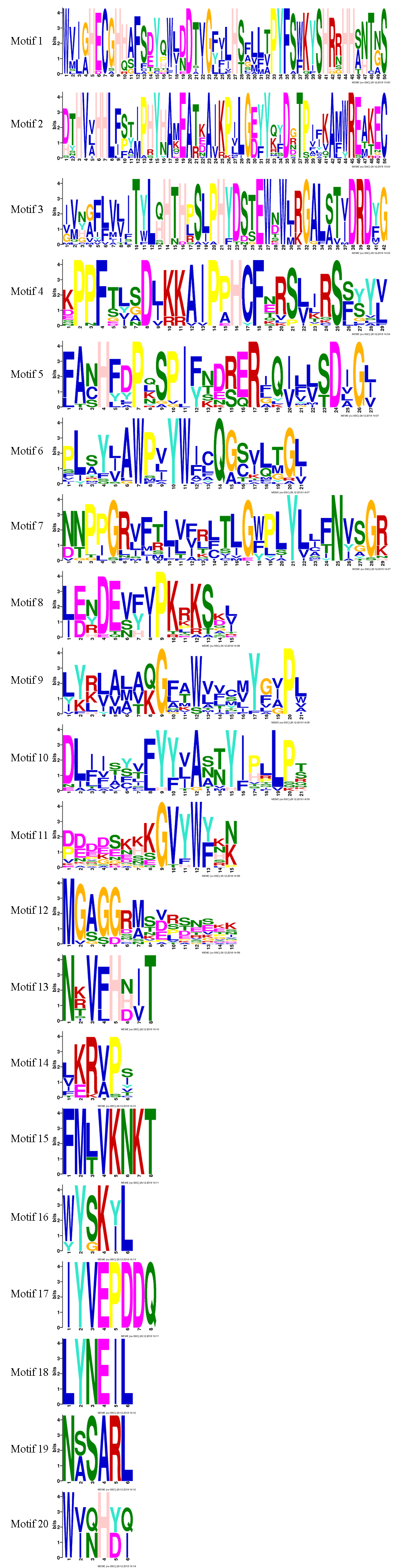


Figure S3. The detailed information of twenty putative conserved motifs.

Supplement: Supplementary file 5 — Additional file 5: Figure S3. The detailed information of putative twenty conserved motifs. [file 12870_2019_2083_MOESM5_ESM.docx]
