# Supplementary material for: Cloning and functional analysis of the FAD2 gene family from desert shrub Artemisia sphaerocephala
Source: BMC Plant Biol. 2019 Nov 8;19:481. doi: 10.1186/s12870-019-2083-5 (PMC6839233; doi:10.1186/s12870-019-2083-5)
Supplement: Supplementary file 4 — Additional file 4: Figure S2. The alignment of the putative polypeptide sequences of AsFAD2 genes together with those of selected ortholog plants. [file 12870_2019_2083_MOESM4_ESM.pdf]

```
AhFAD2-1        ...........MG.AGGRTSVPPSNRTVE....ADYAKRVPHEKPPFSLSQVKKAIPPHCFQRSVIRSFSYVVYDLTIAFCLYYAATNYFH.KLS.....   78
AhFAD2-2        ...........MG.AGGR..VTKIEAQ......KKPLSRVPHSNPPFSVGQLKKAIPPHCFERSLFISFSYVVYDLLVAYLLFYIATTYFH.KLP.....   74
AsFAD2-1        ...........MG.GGGCNVVRKTKSQ..Q...KNPLERVPYEKPPFTISDLKRAIPPHCFQRSVIRSFLSVAYDLIVVFLLYHLTT.YFH.YLP.....   76
AsFAD2-2        ...........MV.SGGSATERSNLESE.......VFKRAPSSQPPFTLSDIRKAIPPHCFNLSLLCSSFYVLVDLLISFSLYYVAAVYIP.RLP.....   75
AsFAD2-4        ...........MG.ASDDMK...............VLERVPISKPPFEYSDLKKAIPPHCFTVSLKRSFYALFHNLFILCTLYYIASNYIH.ILP.....   67
AsFAD2-5        ...........MG.AGGHAIERTNSESE.......FVKRAPSSSPPFTLSDIKKAIPPHCFNRSLLRSSSYLFLDLFFSFFFYYVAAMHIT.RLP.....   75
AsFAD2-6        ...........MG.SGGRATERSNSDCE.......VVKRAPSSKPPFTLSDVKKAIPPHCFNRSLLRSSSYLLVDLFFSILFYYVAATYII.PLQ.....   75
AsFAD2-7        ...........MG.AGGRNSDSPVEAKD.G.....ILKRVPIDPP.FSLSDLKKAIPAHCFERSVIRSSYYVVHDLIVTYVFYFLANTYIP.LLP.....   75
AsFAD2-8        ...........MV.ATDDLK...............LFKRVSISNPPFGYNDLKRAIPPHYFTKSLKCSLYLLSYEILLICTLYYVASNYIH.TLP.....   67
AsFAD2-9        ...........MG.AGGRSNPPDQEVKESD...VGAIKRAPTSKPPFTLADIRKVIPPHCFERPLTRSFSYFFRDLAAISILHYIGSTYIP.QLP.....   79
AsFAD2-10       ...........MG.AGGRNSDPATGGKKTE...TETIERVPYCKPPFTVGDVKKAIPPHCFNRSVIRSFSYVVYDLTIASIFYYLANNYIP.LLP.....   79
AsFAD2-11       ...........MG.AGGQNNALDN.EKD.......VLKRVPISEPPFGIADLKKAVPPHCFKRSLIRSLGYVFRDAIIIYTFYYTASNYFP.LVPNDQ..   77
AsFAD2-13       ...........MG.AGGRNSDSSEE.TK.D.....LLKRVPIEPP.FSLSDLKKAIPAHCFERSVIRSSYYVVHDLIVTYVFYFLANTYIP.LLS.....   74
AsFAD2-15       ...........MG.SGGRATERDNSGNE.......VVKRAPSSKPPFTLADIKKAIPPHCFNRSLIRSSSYLFVDLFFSFLFYYVAATYIV.PLK.....   75
AsFAD2-20       ...........MG.AGGRNSDTSE..GK.N.....ILERVPVDPP.FTLSDLKKAIPAHCFERSVIRSSYYVVHDLIVAYVFYFLANTYIP.SLP.....   73
AsFAD2-21       ...........MG.ASDDMK...............VLERVPISKPPFDYNDLKKAIPPHCFTVSLKRSFYVLFHDLFIVCTLYYIASNYIH.FLP.....   67
AsFAD2-22       ...........MG.AGGRNSDSSEREVK.D.....ILKRVPVDPP.FSLSDLKKAIPAHCFERSVIRSSYYVVHDLIITYVFYFLANTYIP.LLP.....   75
AsFAD2-23       MSKTATLTHRGASAKDSTPTIAANAEKPIITIEGEIVKRVPSLKPNFTIGDIRRAIPEHCFSRPLTKSFGYLFWDLIIVAVLVYATT.FIDLVSDDMFGS   99
AtFAD2          ...........MG.AGGRMPVPTSSKKSE....TDTTKRVPCEKPPFSVGDLKKAIPPHCFKRSIPRSFSYLISDIIIASCFYYVATNYFS.LLP.....   78
BnFAD2-1        ...........MG.AGGRNQVSPPSKKSE....TDTIKRVPCETPPFTVGELKKAIPPHCFKRSIPRSFSYLIWDIIIASCFYYVATTYFP.LLP.....   78
BnFAD2-2        ...........MG.AGGRNQVSPPSKKSE....TDNIKCVPCETPPFTVGELKKAIPPHCFKRSIPRSFSYLIWDIIIASCFYYVATTYFP.LLP.....   78
CaACET          ...........MG.GGGRGRTSQKP..........LMERVSVDPP.FTVSDLKQAIPPHCFKRSVIRSSYYIVHDAIIAYIFYFLADKYIP.ILP.....   71
CoCONJ          ...........MGKGASNKK...............VLERVPITKPPFEYNDLKKAVPPHCFSRPLFRSFYFLLHDIIVTCILFYVASNYIP.MLP.....   68
CpEPOX          ...........MG.AGGRGRTSEKS..........VMERVSVDPVTFSLSELKQAIPPHCFQRSVIRSSYYVVQDLIIAYIFYFLANTYIP.TLP.....   72
CtFAD2-1        ...........MG.GGGCNSASETKAE.EK...KNPLDRVPCAKPPFTISDIKQAIPSHCFNRSLIRSFSYIVYDLAIAFVFYYLATTYIH.RLP.....   78
CtFAD2-2        ...........MG.AGGRNSNPSEGEKKTE...LEGIQRVPYCKPPFTVGDVKKAIPPHCFNRSVIRSFSYVVYDLTIASILYYIATTFIP.LLP.....   79
CtFAD2-3        ...........MG.AGGRMNVAVSGSEKPH.....AFKRVPVSKPPFELSDLKKAVPPHCFKRSLVRSFAALFRDIIIVTALYYLAATIIP.VLP.....   77
CtFAD2-4        ...........MG.AGGRNDVAAS.SEKPH.....AFKRVPVSKPPFELSDLKKAVPPHCFKRSLVRSFAALFRDIIIVSALYYLAATVIP.VLP.....   76
CtFAD2-5        ...........MG.AGGRNNDDATRERD.......VFKHVPVEKPSFGIADLKKAIPPHCFKRSLTTSFYYLFRDLGLIYAFYYIATKYIT.HLP.....   75
CtFAD2-6        ...........MG.AGGRMN.DATTDKD.......VLKRVPTAKTPFEISDLKKAIPPHCFKRSLTTSFYYLFRDICICYTVYHLGSNYLP.LLP.....   74
CtFAD2-7        ...........MG.AGGRMKTE...EKD.......IMKHVPVAKPPFGISDLKKAIPPHCFKRSLIVSFGSFFRDLIVIYSFYYLASTFIP.LLP.....   72
CtFAD2-8        ...........MG.SGGRATERSNPESEV......VVKRAPSSKPPFTLGDIKKAIPPHCFERSLIRSSSYLFLDLFLSSLFYYVAAVYIP.RLP.....   76
CtFAD2-9        ...........MG.AGGRSNPRDLDVKKFDT..VEGIKRAPTSKPPFTLADIKKAIPPHCFQRSLIRSFSYLVYDLTAVSIFYYLATTYIP.QLP.....   80
CtFAD2-10       ...........MG.GGGCIPASKTEAQ..Q...TNHLSRVPYEKPAFTVGDIKRVIPPHCFQRSLIRSFSYVAYDLAIAFLSYHLATTYFH.RLS.....   77
CtFAD2-11_ACET  ...........MG.AGGRNSDSSES..T.D.....ILKRVPIDPP.FTLSDLKKAIPAHCFERSLIRSSYYVVHDLVVAYVFYYLADTYIP.QLP.....   73
DcACET          ...........................................KAIPPHCFEKSLITSFRYLIQDLLMAYALYYVATNYIDQYLP.....   42
DcFAD2_OH       ...........MG.AGGRNSAPSNVKKTET....EALRRAPHEKPPFTIGDLKKAIPAHCFEKSLITSFRYLIQDLLMAYALYYVATNYIDQYLP.....   79
DsACET          ..........................................KKAIPPHCFERSLIRSSYYVVHDLIVAYVFYFLANTYIP.LLP.....   42
FvACET          ...........................................KAIPPHCFEKSLITSFRYLIQDLLMAYALYYVATTYIDQYLP.....   42
GhFAD2-1        ...........MG.AGGRMPIDGIKEEN.....RGSVNRVPIEKPPFTLGQIKQAIPPHCFRRSLLRSFSYVVHDLCLASFFYYIATSYFH.FLP.....   77
GhFAD2-2        ...........MG.AGGRNSVPTSPKKPE....FNSLKRVPYSKPPFTLSEIKKAIPPHCFQRSVLRSFSYLLYDFILASLFYHVATNYFP.NLP.....   78
GmFAD2-1        ...MGLAKETTMG.GRGR..VAKVEVQG.....KKPLSRVPNTKPPFTVGQLKKAIPPHCFQRSLLTSFSYVVYDLSFA.FIFYIATTYFH.LLP.....   82
GmFAD2-2        ...........MG.AGGRTDVPPANRKSE....VDPLKRVPFEKPCFSLSQIKKAIPPHCFQRSVLRSFSYVVYDLTIAFCLYYVATHYFH.LLP.....   78
HaACET          ...........MG.AGGRNSDPSEG..K.N.....ILERVPIDPP.FTLSDLKKAIPAHCFERSVIRSSYYVVHDLIVAYVFYFLANTYIP.LLP.....   73
HaFAD2-1        ...........MG.AGEYTSVTN......E...NNPLDRVPHAKPPFTIGDLKKAIPPHCFQRSLTRSFSYVLSDLTITAVLYHIATTYFH.HLP.....   73
HaFAD2-2        ...........MG.AGGRNSNPVNGEKKPN...PDPLQRVPYCKPPFTVGDVKKAIPPHCFNRSVIRSFSYVVYDLTIASIFYYLANNYIA.LLP.....   79
HaFAD2-3        ...........MG.AGGRNSSP.NGKEKDG...PKPLERALHEKPPFTVGDIKKVIPPHCFKRSVIRSFSYVVYDLTIASIFYYLANNYIP.LLP.....   78
HhACET          ...........MG.AGGRNSETPTAKKTAA....EALRRAPHEKPPFTIGDLKKAVPAHCFEKSLITSFRYLIQDLLMAYSLYYVATNYID.LLP.....   78
NtFAD2          ...........MG.AGGNMSVVTGKTG.EK...KNPLEKVPTSKPPFTVGDIKKAIPPHCFQRSLVRSFSYVVYDLILVSVFYYIAITYFH.LLP.....   78
OeFAD2-1        ...........MG.AGGRLSVPATKAE.EK...KNPLKRVPYLKPPFTVGDIKKTIPPHCFKRSLLRSFSYVVYDLFLVFLFYYIATSYFH.LLP.....   78
OeFAD2-2        ...........MG.AGGRNSIPPEGKKSK....SDVIQRVPQSKPCFSLGDIKKAIPPHCFQRSVLRSFSYVVYDLTIASLLCYVASTYIC.FLP.....   78
RcOH            ...........MG.GGGRNSTVITSNNSEKKGGSSHLKRAPHTKPPFTLGDLKRAIPPHCFERSFVRSFSYVAYDVCLSFLFYSIATNFFP.YIS.....   82
RhACET          ...........................................KAIPPHCFERSVIRSSYYVVHDLIVAYVFYFLANTYIP.LLP.....   41
SiFAD2-1        ...........MG.AGGRNSVPPEGKKSK....SDAFQRVPHSKPPFTLGELKKAIPPHCFQRSIPRSFSYVIYDLAIASLFYYVATNYFH.LLP.....   78
SiFAD2-2        ...........MG.AGGRNSDPTTKDE.QK...KNPLQRVPYAKPPFTLGDIKKAIPPHCFERSVSRSFSYVVYDLVIVFLLYYIATSYFH.LLP.....   78
SlEPOX          ...........MS.DSYDDRMKDHD..........MDERAPIDPAPFSLSDLKKAIPAHCFRRSAVVSSCYVVQDLIITFLLYTVANTYIP.HLP.....   72
Consensus                                                                   p h f       s
```

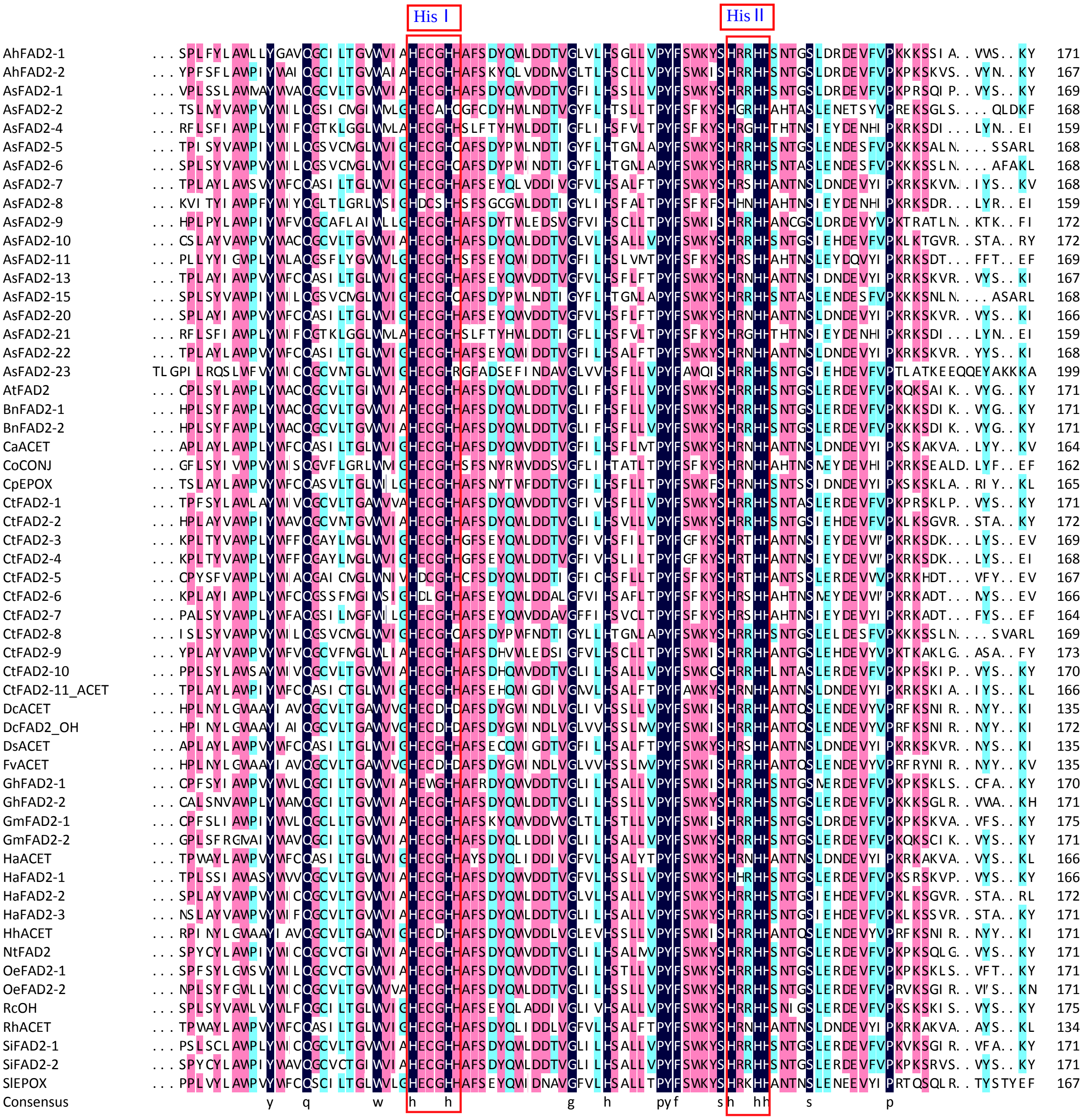
His Ⅰ
His Ⅱ
Consensus

```
AhFAD2-1        LNN............PLGRVLTLTITLTLGVPLYLAFNVSG.RPYDR...FACHYDPYGPIYSD......RERLQIYISDAGVLAVSYGLIRLVMAKGLA 249
AhFAD2-2        MNN............PPGRAISLFITLTLGVPLYLAFNVSG.RPYDR...FASHYDPYAPIYSN......RERLLIYVSDSSVFAVTYLLYHIATLKGLG 245
AsFAD2-1        LNN............PPGRIISLFATLTLGVPLYLAFNVSG.RPYDR...FACHYAPNSPIYNN......RERLQIGLSDIGIITMSFILYRVALVKGLA 247
AsFAD2-2        LNT............PPGRLIRLVILCSIGVVLYICFNVAG.HKYD...KFANHFDPKSPIFND......SERFQILVTDIGLLVTCYGLYKLALACGFT 246
AsFAD2-4        LDN............PIGLVFTILFRLTLGYPSYLIFNYGG.RKYD.E.GLASHLYPCSPIFKD......SQRGQIFVSDVGVFVVLYVYYRVMMTCGAM 238
AsFAD2-5        LNT............PPGRLFRLVILCTVGVLLYVCFNVSG.RKYE...KFANHFDPKSPIYND......RERFQILLTDIGLVVAAYGLYKLALVCGFK 246
AsFAD2-6        LNT............PPGRLFRLVVLCTIGVLLYVCFNVSG.RKYE...KFANHFDPKSPIYNE......RERFQILLTDIGLIVTSYGLYKLALACGFT 246
AsFAD2-7        LNN............PPGRVFTLVFRLTLGFPLYLLTNVSG.KKYE...RFANHFDPLSPIFTE......RERIQVMLSDLGIIAVVYALKLLVAAKGFT 246
AsFAD2-8        IDN............PIGFVFMLFKFILEFPLYLIINYGG.RKY..E.GLACHFYPCSVLFKD......SQRAKVFLSDVGVLVFIYAYYRIMMTCGSI 237
AsFAD2-9        LDN............PIGRSLTLLVKLCLGVYIYLSINAAG.RPYDR...FASHYDPRSPIFSD......SERILIVMSDIGLICSSYLLYKVAMVKGFV 250
AsFAD2-10       LNN............PPGRILTLLVTLTMGVPLYLMFNVSG.RYYDR...FACHFDPNSPIYSN......RERAQIFISDAGILAVMFGLFRLAMVKGLT 250
AsFAD2-11       LNN............GPGNVFTFLARITLGYPLYLLFNVYG.RDYK...RFTNHFLPCSGIFNK......SERGYVVLSDIGIMAVLYALYHLAVTCGGK 247
AsFAD2-13       LNN............PPGRVFTLVFRFTLGFPLYLLTNISG.KKYG...RFANHFDPLSPIFTD......RERIQVLVSDLGLLGVFYAIKLLVAAKGFA 245
AsFAD2-15       LNT............PPGRLFRLVILCSIGVLLYVFFNVSG.RKYD...RFANHFDPKSPIYSD......RERFQILLTDIGLLVASYGVYKVALACGFT 246
AsFAD2-20       LNN............PPGRVFTLVFRFTLGFPLYLLTNISG.KKYG...RFANHFDPLSPIFTD......RERIQVLVSDLGLLGVFYAIKLLVAAKGFA 244
AsFAD2-21       LDN............PIGLVFTILFRLTLGYPSYLIFNYGG.RKYD.E.GLASHLYPCSPIFKD......SQRGQIFVSDVGVFVVLYVYYRVMMTCGAM 238
AsFAD2-22       LNN............PPGRVFTLVFRLTLGFPLYLLTNISG.KKYG...RFANHFDPLSPIFTE......RERIQVVVSDIGILALLYAIKLLVAAKGAA 246
AsFAD2-23       SEHHDDEGFVQQTVGTLSRMFWIVIMLTLGVPAYLIYNVTSNKSYDPS.TWVNHYQPSSPIFTNGLASDAHNCKLILISDVALFAVFAVFYKFIQLTSFT 298
AtFAD2          LNN............PLGRIMMLTVQFVLGVPLYLAFNVSG.RPYDG...FACHFFPNAPIYND......RERLQIYLSDAGILAVCFGLYRYAAACGMA 249
BnFAD2-1        LNN............PLGRTVMLTVQFTLGVPLYLAFNVSG.RPYDGG..FACHFHPNAPIYND......RERLQIYISDAGILAVCYGLFRYAAACGVA 250
BnFAD2-2        LNN............PLGRTVMLTVQFTLGVPLYLAFNVSG.RPYDGG..FACHFHPNAPIYND......RERLQIYISDAGILAVCYGLYRYAAVCGVA 250
CaACET          LNH............PPGRLLIMFITFTLGFPLYLFTNISG.KKYE...RFANHFDPMSPIFKE......RERFQVLLSDLGLLAVLYGVKLAVAAKGAA 242
CoCONJ          LGNN...........PMGLMTMLCKLTFGYAAYIMFNYTG.KKHKSG.GLASHFYPCSPLFND......SERNHVLFSDVGICIVLYACYRIVMVTGAM 243
CpEPOX          LNN............PPGRLLVLIIMFTLGFPLYLLTNISG.KKYD...RFANHFDPMSPIFKE......RERFQVFLSDLGLLAVFYGIKVAVANKGAA 243
CtFAD2-1        LNN............PPGRIISLFATLTLGVPLYLSFNVSG.RPYDR...FACHYAPNSPIYNH......RERLQIWLSDVGIVAMAFVLYRVALVKGVS 249
CtFAD2-2        LNN............PPGRILTLLVTLTLGVPLYLMFNVSG.RYYDR...FACHFDPNSPIYSN......RERAQIFISDAGIFAVLYGLYRLAAVKGLV 250
CtFAD2-3        LNN............PLGSFIVFVFKIVLGFPLYFVFNLYG.RKYE..KGITSHFYPYSPIFND......SERFQIFLTDLGVFGTLYGVYRLALIKGTE 248
CtFAD2-4        LNN............PLGSFVVFVFKIVLGFPLYFVFNLYG.RKYE..KGITSHFYPYSPIFND......SERFQIFLTDLGVFGTLYGVYRLALIKGTE 247
CtFAD2-5        LSN............PVGSFIMLVFRLFFGFPLYFMFNLHG.RIYK...GFPSHFNPLGPIFND......RERANIWLSDAGVLTVVYALYRIGAKEGLQ 245
CtFAD2-6        LNN............PLGNLFMTVVRLLFSFPMYFTFNIHG.RPYN...GFVSHFYPCSPMFND......SERKLVWLSDAGMVAAFYGLYKIAASTSAT 244
CtFAD2-7        LNN............GPGNVFTLLLRTTLGLPLYLIFNTYG.RDYN...GFANHFLPCSGIFND......SERAQVVLSDVGIFAVLYALYRLVLICGLK 242
CtFAD2-8        LNT............PPGRLFRLTILCTVGVLLYICFNVSG.RKYE...KFANHFDPKSPIYND......RERFQILLTDIGLLVTSYGLYKLALACGFA 247
CtFAD2-9        LDN............PIGRTLTLMVKLTLGVYIYLAINAAG.RPYEK...FASHYDPRSEMFSD......NERVLILMSDIGLLSFSFLLYKVAMVCGFA 251
CtFAD2-10       LNN............PPGRLIIVFTTLVLGVPLYLAFNVSG.RPYDR...FACHYAPNSPIFTN......RERLQIWISDAGIIAVSYLLYRMALAKSVA 248
CtFAD2-11_ACET  LNN............PPGRVFTLVFRLTLGFPLYLLTNISG.KKYG...RFANHFDPMSPIFTE......RERIQVLVSDLGILAVIYAIKLAVAAKGAA 244
DcACET          FNN............PPGRVLVWVTTLLIGFPLYLMFNVSG.HKYER...WTSHCDPHSPLYTE......RERKQIIVSDVAILAVIYGLYNLVLAKGFV 213
DcFAD2_OH       LNN............PPGRVIVWLITLLIGFPIYLMFNVSG.HKYER...WTSHYDPHSPLYTE......RERKQIIVSDFAILAVLYGLYNLVLAKGFA 250
DsACET          LNN............PPGRVFTLVFRLTLGFPLYLLTNVSG.KKYE...RFANHFDPMSPIFTE......RERIQVVLSDLGIIAVCYALKVLVAAKGAT 213
FvACET          LNN............PLGRVLVWVTTLLIGFPLYLMFNVSG.HKYER...WTSHYDPHSPLYTE......RERKQIIVSDIAILAVIYGLYRLVLIKGFA 213
GhFAD2-1        LNN............PPGRVLSLVVTLTLGVPMYLAFNVSG.RYYDR...LASHYNPYGPIYSD......RERLQVYISDTGIFAVIYVLYKIAATKGLA 248
GhFAD2-2        FNN............PPGRFLSITIQLTLGVPLYLAFNVAG.RPYDR...FACHYDPYGPIFSD......RERLQIYISDAGVLAVAYALYRLVLAKGVG 249
GmFAD2-1        LNN............PLGRAVSLLVTLTIGVPMYLAFNVSG.RPYDS...FASHYHPYAPIYSN......RERLLIYVSDVALFSVTYSLYRVATLKGLV 253
GmFAD2-2        LNN............PPGRVLTLAVTLTLGVPLYLALNVSG.RPYDR...FACHYDPYGPIYSD......RERLQIYISDAGVLAVVYGLFRLAMAKGLA 249
HaACET          LNN............PPGRVFTLVFRLTLGFPLYLLTNISG.KKYG...RFANHFDPLSPIFTE......RERIQVVISDIGILAVLYATKLLVEAKGAA 244
HaFAD2-1        FNN............TVGRIVSMFVTLTLGVPLYLAFNVSG.RPYDR...FACHYVPTSPMYNE......RKRYQIVMSDIGIVITSFILYRVAMAKGLV 244
HaFAD2-2        LNN............PPGRILTLLVTLTMGVPLYLMFNVSG.RYYDR...FACHFDPNSPIYSN......RERAQIFISDAGILTVLFVLFRVAMTKGLT 250
HaFAD2-3        LNN............PPGRILTLLVTLTMGVPLYLMFNVSG.RYYDR...FACHFDPNSPIYSN......RERAQIFISDAGILTVFYILFRLASTKGLV 249
HhACET          LNN............PPGRVFVWLSTLLIGFPLYLMFNVSG.HKYER...WTSHYDPHSPLYSD......RERKEIIISDVAILTVIYGLYRLVLAKGFE 249
NtFAD2          LNN............PPGRVMSLTVTLTLGVPLYLAFNVSG.RHYDR...FACHYDPYGPIYND......RERLQIFLSDAGVLGAGYLLYRIALVKGLA 249
OeFAD2-1        LNN............PPGRVMTLVITLTLGVPLYLALNVSG.RPYDR...FACHYDPHGPIYND......RERLQIYISDVCVIATSYILYRVALACGLV 249
OeFAD2-2        LNN............PPGRVLLLVFQLTLGVPLYLMFNVSG.RHYDR...FACHYNPHSPMYSD......RERAQILISDAGVLAVTCGLYRLTAAKGLA 249
RcOH            SNN............PPGRVLTLAATLLLGVPLYLAFNVSG.RPYDR...FACHYDPYGPIFSE......RERLQIYIADLGIFATTFVLYQATMAKGLA 253
RhACET          LNX............PPGRVFTLVFRLTLGFPLYLLTNISG.KKYG...RFANHFDPLSPIFTD......RERVQVLLSDLGLLAAFYAIKLLVAAKGFA 212
SiFAD2-1        MNN............PPGRVLMLIVQFTLGVPLYLLFNVSG.RHYDR...FACHYDPKSPIYSD......RERLQILISDAGVLAVTYGLYRLAIAKGLA 249
SiFAD2-2        LNN............PLGRVITLVVTLTLGVPLYLLFNVSG.RPYNR...FACHFDPYGPIYND......RERLQIFISDAGIIAAVCVLYRVALVKGLA 249
SlEPOX          LDN............TPGRILILVIMLTLGFPLYLLTNVSG.KKYD...RFTNHFDPLSPIFTE......RERIQVALSDLGIVAVFYGLKFLVQTKGFG 245
Consensus                                        y   n               h  p                      d
```

His III

```
AhFAD2-1        VVVAVYGVPLLVVNGFLVLITYLQHTHPALPHYDSSEWDWLRGALATVDRDYG.ILNKVFHNITDTHVAHHLFSTMPHYHAMEATNAIKPILGEYYRFDG   348
AhFAD2-2        VVVCVYGVPLLIVDGFLVTITYLQHTHASLPHYDSSEWDWLRGALATVDRDYG.ILNKAFHHITDTHVAHHLFLTMPHYHAMEATNAIKPILGDYYCIDG   344
AsFAD2-1        VVISVYGIPLMVNGFLVLITYLQHTHPSLPHYDNSEWEWLKGALATVDRDYG.VLNKVFHNITDTHVVHHLFSTMPHYNAMEATEAVRPLLGEYYRLDA    346
AsFAD2-2        VLVMYFAPLVVLYAYVAVITVLNHTHSSLPHYDSTEWNWLKGGLSTMDRDYG.ILNSVFHHITDAHVVHHLFPTIAHYHAMEATKAIKPILGEYYHSDN    345
AsFAD2-4        VAFYVYGVPVFIMGAILMVFTYLQHNHPSIPHYDSTEWTWMRGALSVVDRDMG.FM..LVKNKTDYHVVHHLFPAIPRYHACEATEAIKPILGDYYKYDG   335
AsFAD2-5        VLVMVYFAPLVIVYGFLVVITVLHHTHRSLPHFDSTEWSWLRGALATVDRDYG.VFNTVLHHITDTHVAHHLFFTIPHYNAMEATKAIKPFLGEYYCFDD   345
AsFAD2-6        VLVLIYFAPLVIVYGFLVVITVLHHTHRSLPHYDSTEWNWLRGALSTIDRDYG.ILNTVFHHITDTHVAHHLFTIPHYYAMEATNAIKPILGEYYCYDD    345
AsFAD2-7        VVLCMYGIPVIGVHAFFVLITYLHHTHLSLPHYDSTEWKWIKGALSTIDRDFG.FLNRVFHDVTHTHVLHHLISYIPHYHAKEARDAIKPVLGDYYKIDR   345
AsFAD2-8        VVFNLYGGPVVIMCGVLLVLTYLQHTHPSIPHYDSTEWSWIRGALSTVDRDVG.FM..TVKNKTDNHVVHHLFPAIPSYHAHEATEAIKPILGDYYNYDG   334
AsFAD2-9        VLFCFYVGPITVMNAFLVTITYLHHTHPSLPHFDDSEWNWMKGAFATVDRDYG.VLNKVFHNITDTHVLHHLFSYIPHYHAMEATNAIKPMLGEFYCIDN   349
AsFAD2-10       VVLTMYAGPLLVVNGFLVLITFLQHTHPSLPHYDSTEWDWLRGALATVDRDYG.ILNKVFHNITDTHVAHHLFSTMPHYHAMEATKAIKPLLGEYYCFDG   349
AsFAD2-11       STLFLFVIPLFMCGGFFILSTYLSHTHPSVAHYDSTEWDWLRGALSTIDRDFG.ILNWQHDINSSHVIHHLFPSMPHYHAVEATKAVRPILGEYYMSDN    346
AsFAD2-13       VVTSMYLIPALGVHVFFVIITYLHHTHLSLPHYDSTEWNWIRGALSTIDRDFG.FLNRVFHDVTHTHVLHHLISYIPHYHAKEARDAIKPVLGEFYKIDR   344
AsFAD2-15       VIVMVYFAPLVIVYGFLVVITVLHHTHRSLPHYDSTEWNWLRGALSTIDRDYG.ILNTVLHHITDTHVAHHLFFTIPHYNAMEATKAIKPILGEYYCFDD   345
AsFAD2-20       VVTSMYLIPALGVHVFFVIITYLHHTHLSLPHYDSTEWNWIRGALSTIDRDFG.FLNRVFHDVTHTHVLHHLISYIPHYHAKEARDAIKPVLGEFYKIDR   343
AsFAD2-21       VAFYVYGVPVFIMGAILMVFTYLQHNHPSIPHYDSTEWTWMRGALSVVDRDMG.FM..LVKNKTDYHVVHHLFPAIPRYHACEATEAIKPILGDYYKYDG   335
AsFAD2-22       VVACMYGVPVLGVHMFFVLITYLHHTHLSLPHYDSTEWNWIRGALSTIDRDFG.FLNRVFHDVTHTHVLHHLISYIPHYHAKEARDAIKPVLGEFYKIDR   345
AsFAD2-23       YFLYIYFIPYLVVNAVLVLITYLQHTDLALPHYTDGEWDWLRGALATIDRDYG.FLNIVLHHITDTHVVHHLFSFMPHYHAEEATVAVKKLIGDYYRYDS   397
AtFAD2          SMCLYGVPLLIVNAFLVLITYLQHTHPSLPHYDSSEWDWLRGALATVDRDYX.ILNKVFHNITDTHVAHHLFSTMPHYNAMEATKAIKPILGDYYCFDG    348
BnFAD2-1        SMVCFYGVPLLIVNGLLVLITYLQHTHPSLPHYDSSEWDWLRGALATVDRDYG.ILNKVFHNITDTHVAHHLFSTMPHYHAMEATKAIKPILGEYYCFDG   349
BnFAD2-2        SMVCFYGVPLLIVNGFLVLITYLQHTHPSLPHYDSSEWDWLRGALATVDRDYG.ILNKVFHNITDTHVAHHLFSTMPHYHAMEATKVIKPILGEYYCFDG   349
CaACET          VVTCIYGIPVLGVFIFFDIITYLHHTHLSLPHYDSSEWNWLRGALSTIDRDFG.FLNSVLHDVTHTHVMHHLFSYIPHYHAKEARDAINTVLGDFYKIDR   341
CoCONJ          SAFYVYGIPVVIMSAILFAATYLQHTHPSIPHYDTTEWNWLRGALSTIDRDLG.FF..NMN.KTHYHVIHHLFPVIPEYHACEATEAIKPILGCYYKYDG   339
CpEPOX          VVACMYGVPVLGVFTFFDVITFLHHTHCSSPHYDSTEWNWIRGALSAIDRDFG.FLNSVFHDVTHTHVMHHLFSYIPHYHAKEARDAIKPILGDFYMIDR   342
CtFAD2-1        VVVCVYGIPLLIVNGFLVLITFLQHTHPSLPHYDGSEWDWLRGALATVDRDYG.VLNKVFHNITDTHVVHHLFSTMPHYHAMEATRAVKGLLGEYYCFDE   348
CtFAD2-2        VVLTVYAGPLLVVNGFLVLITFLQHTHPSLPHYDSTEWDWLRGALATIDRDYG.ILNKVFHNITDTHVTHHLFSTMPHYHAMEATKAIIPILGDYYCFDG   349
CtFAD2-3        VVINFYGMPILFMSGFFILLTYLHHTHPSIPHYDSTEWDWLRGALATVDRNFG.FLNHAFHDVTRTHAVHHLFPTIPHYHTFEARQAVMPILGDYYKYDD   347
CtFAD2-4        VVINFYGMPILFMSGFFILLTYLHHTHPSIPHYDSTEWDWLRGALATVDRNFG.FLNHAFHDVTRTHAVHHLFPTIPHYHTFEARQAVMPILGDYYKYDD   346
CtFAD2-5        SVLFVYIYPLMAMSAFFIMFTYLHHTHPSIAHYDSSEWDWLRGALSTVDRDYG.ILNNIFHDVTSAHVVHHLFSSIPHYNTVEATQYIKPILGDYYNYDY   344
CtFAD2-6        VLFCIYGAPLLVMNAHFIFFTFLHHSHVSLAHYDSREWDWIRGALSTVDRNYG.ILNTVFHDVTCAHVVHHLISTIPHYHTVEATNAVKPILGDYYKYDD   343
CtFAD2-7        STIFLQGIPLFVMSGFFIFLTYLNHTHPAIAHYDSTEWDWLRGALSTIDRDFG.ILNRVFHNANHTHGIHHLFPTIPHYHAIEAREAVKPILGDYYMYDD   341
CtFAD2-8        VLITIYFAPLVIVYGFLVVITVLHHTHRSLPHYDSTEWNWLRGALSTMDRDYG.VFNTVLHHITDTHVAHHLFFTIPHYNAMEATKAIKPFLGEYYCFDD   346
CtFAD2-9        VVFCVYGGALMVMNAFLVTITYLHHTHPSLPHYDDSEWNWMKGAFATVDRDYGVVLNKVFHNITDTHVLHHLFSYIPHYHAMEATKAIRPVLGEFYCIDR   351
CtFAD2-10       VVVCIYGVPLLIVNGFLVMITYLQHTHPSLPHYDDSEWDWLRGALATVDRDYG.LLNKVFHNITDTHVVHHLFSTMPHYHAMEATKAVEPVLGGYYRFDD   347
CtFAD2-11_ACET  VVISMYGIPVIGVHVFFVLITYLHHTHLSLPHYDSTEWNWIRGALSTIDRDFG.FLNRVFHDVTHTHVLHHLISYIPHYHAKEARDAIKPILGEYYKIDR   343
DcACET          VVFCVYGGPLLVVNGVFTLITILNHTHPSLPYYDSTEWDWLRGALCTVDRDYG.ILNKVFHNVCNAHVCHHIFSMPHYHGLEATEAMKPLLGDYYCYDG    312
DcFAD2_OH       VVFCVYGGPLLVVNGVFTLITILNHTHPSLPYYDSSEWDWLRGALCTVDRDYG.ILNKVFHNVCNAHVCHHIFSMPHYHGLEATEAMKPLLGDYYCYDG    349
DsACET          VVMCMYGVPVIGVHAFFVLITYLHHTHLSLPHYDSSEWNWIKGALSTIDRDFG.FLNRVFHDVTHTHVLHHLISYIPHYHAKEARDAIIPVLGEFYKIDR   312
FvACET          VVFCVYGGPLLVVNMVFTLITILNHTHPSVPYYDSTEWDWLRGALCTVDRDYG.ILNKVFHNVCNAHVCHHIFSMPHYHGLEATEAMKPVLGDYYCYDG    312
GhFAD2-1        VLLCTYGVPLLIVNAFLVLITYLQHTHSALPHYDSSEWDWLRGALSTMDRDFG.VLNKVFHNITDTHVAHHLFSTMPHYHAMEATKAIKPILGKYYPFDG   347
GhFAD2-2        VVISVYGVPLLVVNAFLVMITYLQHTHPSLPHYDSSEWDWMRGALSTVDRDYG.ILNKVFHNITDTHVAHHLFSTMPHYHAMVATKAIKPILGEYYCFDG   348
GmFAD2-1        VLLCVYGVPLLIVNGFLVTITYLQHTHFALPHYDSSEWDWLKGALATMDRDYG.ILNKVFHHITDTHVAHHLFSTMPHYHAMEATNAIKPILGEYYCFDD   352
GmFAD2-2        VVVCVYGVPLLVVNGFLVLITFLQHTHPALPHYTSSEWDWLRGALATVDRDYG.ILNKVFHNITDTHVAHHLFSTMPHYHAMEATKAIKPILGEYYRFDE   348
HaACET          VVTCMYLIPVLGVHMFFVLITYLHHTHLSLPHYDSSEWNWIRGALSTIDRDFG.FLNRVFHDVTHTHVLHHLISYIPHYHAKEARDAIKPVLGEFYKIDR   343
HaFAD2-1        VVICVYGVPLMVVNAFLVLITYLQHTHPGLPHYDSSEWEWLKGALATVDRDYG.VLNKVFHHITDTHVVHHLFSTMPHYNAMEACKALRPVLGEYYRFDK   343
HaFAD2-2        VVLTMYAGPLLVVNGFLVLITFLQHTHPSLPHYDSTEWDWLRGALATIDRDYG.VLNKVFHNITDTHVAHHLFSTMPHYHAMEATKVIKPILGEYYCFDG   349
HaFAD2-3        VVLTMYGGPLLVVNGFLVLITFLQHTHPSLPHYDSTEWDWLRGALATVDRDYG.ILNKVFHNITDTHVTHHLFSTMPHYHAMEATKAIKPILGDYYCFDG   348
HhACET          VVFLVYGGPLLVVNGVFVLITILNHTHPSLPYYDSTEWDWLRGALCTVDRDYG.ILNKVFHNVCNAHVCHHIFSMPHYHGLEATQAMKPILGEYYCYDG    348
NtFAD2          VLVCMYGVPLLIVNGFLVLITYLQHTHPSLPHYDSSEWDWLRGALATVDRDYG.ILNKVFHNITDTHVVHHLFSTMPHYNAMEATKAVKPLLGDYYCFDG   348
OeFAD2-1        VLTCVYGVPLLIVNGFLVLITYLQHTHPPLPHYDSSEWDWLRGALATVDRDYG.VLNNVFHNITDTHVAHHLFSTMPHYHAMEATKAIKPLLGEYYCSDG   348
OeFAD2-2        VVFCVYGGPLLVVNGFLVLITYLQHTHPSLPHYDSSEWDWLRGALATVDRDWG.ILNKVFHNITDTHVSHHLFSTMPHYHAMEATKAIKPILGNYYCFDG   348
RcOH            VVMRIYGVPLLIVNCFLVMITYLQHTHPAIPRYGSSEWDWLRGAMVTVDRDYG.VLNKVFHNIADTHVAHHLFATVPHYHAMEATKAIKPIMGEYYRYDG   352
RhACET          VVTSMYLIPVMGVHMFFVLITYLHHTHLSLPHYDSTEWNWIKGALSTIDRDFG.FLNRVFHDVTHTHVLHHLISYIPHYHAKEARDAIKPVLGEFYKIDR   311
SiFAD2-1        VLVCVYGGPLLVVNAFLVLITYLQHTHPALPHYDSSEWDWLRGALATVDRDYG.ILNTVFHNITDTHVAHHLFSTMPHYHAMEATKAIKPILGNYYCFDG   348
SiFAD2-2        VLVCVYGVPLLIVNGFLVLITFLQHTHPSLPHYDSSEWDWLRGALATVDRDYG.VLNKVFHNITDTHVTHHLFSTMPHYHAMEATKAIKPILGCYYCFDG   348
SlEPOX          VVMCMYGVPVIGLNSFIIVITYLHHTHLSSPHYDSTEWNWIKGALTTIDRDFG.LLNRVFHDVTHTHVLHHLFPYIPHYHAKEASEAIKPILGDYRMDR    344
Consensus                            t l h        ew w g      dr                h   hh      y   a        g    d
```

```
AhFAD2-1         TPFYKAMWREAKECLYVESDQG......AQ...NKGVFWYNNKL.   383
AhFAD2-2         TPFYKALWREAKECLYVEPDDG......AS...KKGVYWYKNKF.   379
AsFAD2-1         TPFYVAMWREAKECLFVEPD........EG...KKGVFWYKNKM.   379
AsFAD2-2         TSVIKAMWREATECFYVEADE...SGSN.....KKGVYWFKNKL.   381
AsFAD2-4         TPAIQAFWREMKGCIYVEPDDCDNDKNK.....KSGVYWFKK...   372
AsFAD2-5         TPIIKAMWREATECFFVEADE...GEDK.....SKGVYWFNNKM.   381
AsFAD2-6         TPIIKAMWREATECFFVEADE...GNN.......KGVYWFNNKM.   379
AsFAD2-7         TPIFKAMWREAKECMYIEPDQD....SE.....HKGVYWYHKL..   379
AsFAD2-8         TPILKAFWREMKECVYVESDNYDDDKKK.....SSGVYWFRK...   371
AsFAD2-9         TPFWVALWRETKNCLFIEPDEV......DE..KNKGVYWYSNKY.   385
AsFAD2-10        TSVFKAMYRETKECIYVDKDEE......AK....EGVYWYRNKV.   383
AsFAD2-11        TPPLKAFWRETKECIYAE.PD...DSSE.....HKGVYWFSK...   379
AsFAD2-13        TPIFKAMWREAKECIYIEPDEG....SK.....HEGVYWYHKM..   378
AsFAD2-15        TPIVKAMWREATECFFVEADE...ADSD.....SKGVYWFNNKM.   381
AsFAD2-20        TPIFKAMWREAKECIYIEPDEG....SK.....HQGVYWYHKM..   377
AsFAD2-21        TPAIQAFWREMKECIYVEPDDCDNDKNK.....KSGVYWFKK...   372
AsFAD2-22        TPIFKAMWREAKECIYIEPDED....SE.....HKGTYWYHKM..   379
AsFAD2-23        TPIATALWRSFRDCIAVHPDEN...........CKGVHWFLPK..   429
AtFAD2           TPWYVAMYREAKECIYVEPDRE......GD...KKGVYWYNNKL.   383
BnFAD2-1         TPVVKAMWREAKECIYVEPDRC......GE...KKGVFWYNNKL.   384
BnFAD2-2         TPVVKAMWREAKECIYVEPDRC......GE...KKGVFWYNNKL.   384
CaACET           TPILKAMWREAKECIFIEPEKG....RE.....SKGVYWYNKF..   375
CoCONJ           TPFLKALWREMKDCIYVESDQ....GQK.....KQGIYWFKNKI.   374
CpEPOX           TPILKAMWREGRECMYIEPDS......K.....LKGVYWYHKL..   374
CtFAD2-1         TPFYAAMWREAKECLFVEAD........EG...KGGVFWYKNK..   380
CtFAD2-2         TSVFKAMYRETKECIYVDKDEE......VK....DGVYWYRNKI.   383
CtFAD2-3         TPILQAIWRETKDCIFIEPEE...VNGE.....KKGIYWFYK...   381
CtFAD2-4         TPILEAVWRETKDCIFIEPEE...VNGE.....KKGIYWFYK...   380
CtFAD2-5         TPILKAIWRDTKECLFIE......EDPE.....KKGVYWFHK...   375
CtFAD2-6         TPILKAFWRETKNCIYVEPD....EGAE.....DSGVYWFRR...   376
CtFAD2-7         TPILKAMWRDTKECIYVE.PD...D..E.....KKGVYWYFK...   372
CtFAD2-8         TPIIKAMWREATECFFVEADE...GEGK.....SKGVYWFNNKM.   382
CtFAD2-9         TPFFVALWRESKSCLFIEPDES......DE..KNKGIYWYRSKY.   387
CtFAD2-10        TPFYVAMWREAKECLYVETD........EK...KGGVFWYKNKY.   380
CtFAD2-11_ACET   TPIFKAMWREAKECIYIEPDED....SE.....HKGTYWYHKM..   377
DcACET           TPILKAMYREMK.................................   324
DcFAD2_OH        TPILKAMYREMKECIYVEKDEG......ET....KGVYWSRKDI.   383
DsACET           TPIFKAMWREAKEC...............................   326
FvACET           TPILKAMYREMK.................................   324
GhFAD2-1         TPIYKAMWREAKECLYVEPDVG......GGGGGSKGVFWYRNKF.   385
GhFAD2-2         MPVYKAIWREAKECLYVEPDEG......DK...DKGVFWFRNKL.   383
GmFAD2-1         TPFYKALWREARECLYVEPDEG......TS...EKGVYWYRNKY.   387
GmFAD2-2         TPFVKAMWREARECIYVEPDQS......TE...SKGVFWYNNKL.   383
HaACET           TPIFKAMWREAKECIYIEPDED....SE.....HKGTYWYHKM..   377
HaFAD2-1         TPFYVAMWREMKECLFVECDD.......EG...KGGVFWYKNKMN   378
HaFAD2-2         TSIFKAMYRETKECIYVDKDEE......VK....DGVYWYRNKI.   383
HaFAD2-3         TSIFKAMYRETKECIYVDKDED......VK....DGVYWYRNKI.   382
HhACET           TPILKAMYREMKECIYVEKDEG......ET....KGVYWYRSEF.   382
NtFAD2           TPVFKAMWREAKECIYVEKDEA......SQ...GKGVFWYKNKL.   383
OeFAD2-1         TPFYKAMWREAKECLYVEPDE........P...NKGVFWYKNKF.   381
OeFAD2-2         TPIFKAMWREAKECIYVERDEG......DQ...NKGVFWYNNTL.   383
RcOH             TPFYKALWREAKECLFVEPDEG......AP...TQGVFWYRNKY.   387
RhACET           TPIFKAMWRXAKEXV..............................   326
SiFAD2-1         TPIFKAVWREAKECIYVEPDEG......DK...NKGVFWYNNKL.   383
SiFAD2-2         TPFYKAMWREAKECLYVEPDES......TP...DKGVFWYKNKF.   383
SlEPOX           TPFFKAMWREAKECIYIECDAD....SK.....HKGTYWYHKM..   378
Consensus             a  r
```
