# Supplementary material for: Cloning and functional analysis of the FAD2 gene family from desert shrub Artemisia sphaerocephala
Source: BMC Plant Biol. 2019 Nov 8;19:481. doi: 10.1186/s12870-019-2083-5 (PMC6839233; doi:10.1186/s12870-019-2083-5)
Supplement: Supplementary file 6 — Additional file 6: Table S3. Fatty acid composition of transgenic yeast cells. [file 12870_2019_2083_MOESM6_ESM.docx]

Table S3. Fatty acid composition of transgenic yeast cells.

| Samples | Fatty acid composition and content (% w/w) | | | | | |  | Conversion ratio (%) | |
| --- | --- | --- | --- | --- | --- | --- | --- | --- | --- |
|  | C16:0 | C16:1 | C16:2 | C18:0 | C18:1 | C18:2 |  | C16:1→C16:2 | C18:1→C18:2 |
| pYES2 | 10.50±0.09 | 56.91±0.54 | – | 3.93±0.15 | 28.66±0.30 | – |  | – | – |
| AsFAD2-1 | 12.41±0.04 | 31.61±0.23 | 18.10±0.03 | 6.95±0.05 | 12.35±0.12 | 18.58±0.06 |  | 36.41 | 60.07 |
| AsFAD2-2 | 12.41±0.10 | 55.23±0.44 | – | 4.45±0.09 | 27.90±0.26 | – |  | – | – |
| AsFAD2-4 | 11.58±0.01 | 55.43±0.46 | – | 4.77±0.11 | 28.22±0.33 | – |  | – | – |
| AsFAD2-5 | 12.88±0.03 | 57.19±0.12 | – | 4.43±0.01 | 25.50±0.15 | – |  | – | – |
| AsFAD2-6 | 15.04±0.03 | 53.72±0.17 | – | 5.00±0.08 | 26.24±0.06 | – |  | – | – |
| AsFAD2-7 | 11.83±0.06 | 56.03±0.52 | – | 5.07±0.12 | 27.06±0.34 | – |  | – | – |
| AsFAD2-8 | 12.96±0.01 | 54.68±0.51 | – | 4.60±0.11 | 27.77±0.40 | – |  | – | – |
| AsFAD2-9 | 11.10±0.08 | 57.32±0.57 | – | 4.05±0.11 | 27.52±0.38 | – |  | – | – |
| AsFAD2-10 | 12.77±0.11 | 42.91±0.54 | 9.95±0.04 | 5.60±0.11 | 12.23±0.25 | 16.54±0.25 |  | 18.82 | 57.49 |
| AsFAD2-11 | 11.90±0.04 | 55.39±0.46 | – | 4.72±0.08 | 27.99±0.35 | – |  | – | – |
| AsFAD2-13 | 12.41±0.00 | 56.51±0.05 | – | 4.91±0.01 | 26.16±0.05 | – |  | – | – |
| AsFAD2-15 | 12.02±0.00 | 59.70±0.18 | – | 3.45±0.03 | 24.83±0.15 | – |  | – | – |
| AsFAD2-20 | 12.72±0.01 | 56.06±0.16 | – | 5.00±0.04 | 26.09±0.20 | – |  | – | – |
| AsFAD2-21 | 12.12±0.03 | 56.72±0.01 | – | 3.88±0.01 | 27.27±0.04 | – |  | – | – |
| AsFAD2-22 | 11.66±0.02 | 55.77±0.10 | – | 4.90±0.05 | 27.66±0.03 | – |  | – | – |
| AsFAD2-23 | 12.90±0.00 | 55.91±0.09 | – | 5.45±0.05 | 22.45±0.04 | 3.29±0.00 |  | – | 12.78 |

Note: Data represents the mean ± SE of triplicate measurements. Conversion ratio (%) = products / (products + substrates) ×100. “–”, not detected.
