# Supplementary material for: Cloning and functional analysis of the FAD2 gene family from desert shrub Artemisia sphaerocephala
Source: BMC Plant Biol. 2019 Nov 8;19:481. doi: 10.1186/s12870-019-2083-5 (PMC6839233; doi:10.1186/s12870-019-2083-5)
Supplement: Supplementary file 8 — Additional file 8: Table S5. Primers used in the 5’RACE of twenty-one AsFAD2 genes in A. sphaerocephala. [file 12870_2019_2083_MOESM8_ESM.docx]

Table S5. Primers used in the 5´RACE of twenty-one *AsFAD2* genes in *A. sphaerocephala*.

| Primer gene | Outside primer | Inside primer |
| --- | --- | --- |
| *AsFAD2-1* | GATTACGCCAAGCTTTTTGGTCCACAGACATAAAC | GATTACGCCAAGCTTGTCTGGCTCCACGAAAAG |
| *AsFAD2-2* | GATTACGCCAAGCTTCCCCATTATGACTCCACCGA | GATTACGCCAAGCTTTTGAGGCTGACGAAAGTGGC |
| *AsFAD2-4* | GATTACGCCAAGCTTGTAAAACAATGTGGCGGTAT | GATTACGCCAAGCTTACTCGTTCCAAAACCTTCAT |
| *AsFAD2-5* | GATTACGCCAAGCTTGTCGGTAATGTGATGAAGAAC | GATTACGCCAAGCTTCGATAACCAAAGGAGCAAAA |
| *AsFAD2-6* | GATTACGCCAAGCTTTTGATAACTTACAGGTATGC | GATTACGCCAAGCTTGCCCCGTCGTCAAAACCACC |
| *AsFAD2-7* | GATTACGCCAAGCTTTCATGCAAAGGAAGCGAGGG | GATTACGCCAAGCTTTCGAGCCAGATCAAGACAGC |
| *AsFAD2-8* | GATTACGCCAAGCTTAGCACCTCTAATCCAACTCCAC | GATTACGCCAAGCTTGAATGAAAAGTATGGGGTGAGA |
| *AsFAD2-9* | GATTACGCCAAGCTTAAAACAATGGGGTGGTATGA | GATTACGCCAAGCTTTCGGATTCCTTGACTTCTTG |
| *AsFAD2-10* | GATTACGCCAAGCTTACAAGGAGGGTGAGGATTCTGC | GATTACGCCAAGCTTATAGGCGAGTGACTGAGGGAGG |
| *AsFAD2-11* | GATTACGCCAAGCTTTTTGAACAATGGACCAGGGA | GATTACGCCAAGCTTTCTACGCCGAACCTGACGAT |
| *AsFAD2-12* | GATTACGCCAAGCTTCTCCTTTCACCATCAGCGACCT | GATTACGCCAAGCTTTGACCGTTTCGCCTGCCACTAC |
| *AsFAD2-13* | GATTACGCCAAGCTTCGTTTAAGAAGGTCGTCTTTAGTCT | GATTACGCCAAGCTTGTGATGAGTGGAAGTTGTTAGT |
| *AsFAD2-14* | GATTACGCCAAGCTTAAAGAGCAGAATGAAGGACGAA | GATTACGCCAAGCTTAGAGGAGTAGGGAGAAGAGGAA |
| *AsFAD2-15* | GATTACGCCAAGCTTTGGAGCATAAAATCACAAGC | GATTACGCCAAGCTTAAGGAGCAAGCATACCAGTG |
| *AsFAD2-16* | GATTACGCCAAGCTTCATCACTCGCCTACCAACATCC | GATTACGCCAAGCTTTCACCGTCGTCACCATTCTAAC |
| *AsFAD2-19* | GATTACGCCAAGCTTAGGTGAAGACAAAAGCAAAGGT | GATTACGCCAAGCTTTTGTTCTACCACCTTCCCTTCT |
| *AsFAD2-20* | GATTACGCCAAGCTTGAAGGTAGCAAGCACCAA | GATTACGCCAAGCTTATAAGGGTTGTGCTTGGCTC |
| *AsFAD2-21* | GATTACGCCAAGCTTCAACGAGATTCTTGACAACCCA | GATTACGCCAAGCTTTTGTTTCCTGCCATTCCACGCT |
| *AsFAD2-22* | GATTACGCCAAGCTTCGGGAACTCCATACATACACGCTA | GATTACGCCAAGCTTGAAGAGTGCGGAATGGAGGATG |
| *AsFAD2-23* | GATTACGCCAAGCTTGCGATGATGGTTGATAATGGT | GATTACGCCAAGCTTGTTGTAGATAAGATAAGCCGG |
| *AsFAD2-24* | GATTACGCCAAGCTTGGATGGCTCTTGTATGTTTGTT | GATTACGCCAAGCTTGTGTATGGGTTCTTGGTTGTCA |

Note: Underlined bases were added according to the instructions of SMARTer^®^ RACE 5′/3′ Kit.
