# Supplementary material for: Cloning and functional analysis of the FAD2 gene family from desert shrub Artemisia sphaerocephala
Source: BMC Plant Biol. 2019 Nov 8;19:481. doi: 10.1186/s12870-019-2083-5 (PMC6839233; doi:10.1186/s12870-019-2083-5)
Supplement: Supplementary file 9 — Additional file 9: Table S6. Primers used in the 3’RACE of twenty-one AsFAD2 genes in A. sphaerocephala. [file 12870_2019_2083_MOESM9_ESM.docx]

Table S6. Primers used in the 3´RACE of twenty-one *AsFAD2* genes in *A. sphaerocephala*.

| Primer gene | Outside primer | Inside primer |
| --- | --- | --- |
| *AsFAD2-1* | GATTACGCCAAGCTTGGAGCCAGACGAGGGGAAAA | GATTACGCCAAGCTTGTGTTTAGGCATTGTATGAGTTGTG |
| *AsFAD2-2* | GATTACGCCAAGCTTTCTCCTAAGATGGGTTTGATTG | GATTACGCCAAGCTTTATTTGGAAACGCTCACTATCG |
| *AsFAD2-4* | GATTACGCCAAGCTTAGAAAGCAATACCGCCACAT | GATTACGCCAAGCTTTAGGCGGATTGTGGATGTTG |
| *AsFAD2-5* | GATTACGCCAAGCTTCGTTTATGGGTTCCTTGTGGTC | GATTACGCCAAGCTTTTCTTCATCACATTACCGACAC |
| *AsFAD2-6* | GATTACGCCAAGCTTGGTGGTTTTGACGACGGGGC | GATTACGCCAAGCTTCGATTCTCTAAAAAGGAACC |
| *AsFAD2-7* | GATTACGCCAAGCTTCACATTGCTTTGAAGATTGG | GATTACGCCAAGCTTTTGATTGCGTCCCTCGCTTCC |
| *AsFAD2-8* | GATTACGCCAAGCTTTATCCCTAAACGCAAGTCAG | GATTACGCCAAGCTTACAAGGCTCAATCTGGGTAT |
| *AsFAD2-9* | GATTACGCCAAGCTTCGGTGGTCGTTCAAATCCTC | GATTACGCCAAGCTTGGAAGGTCATACCACCCCATT |
| *AsFAD2-10* | GATTACGCCAAGCTTTGTCAAGAAAGCCATCCCACCC | GATTACGCCAAGCTTGTATGCTGGACCCTTGCTTGTG |
| *AsFAD2-11* | GATTACGCCAAGCTTCAATAAACACCTTTGTGCTCCG | GATTACGCCAAGCTTCGCCTCTTAGCCAATCCCACT |
| *AsFAD2-12* | GATTACGCCAAGCTTGAAGCCCACAGTGTCGTCAACC | GATTACGCCAAGCTTGTGTTTGTGCGAAGAAGTGGTT |
| *AsFAD2-13* | GATTACGCCAAGCTTCTCTTTCAAGGTTCACTTCC | GATTACGCCAAGCTTTCACATTCAGAATTATGGGTGC |
| *AsFAD2-14* | GATTACGCCAAGCTTCATTCTTTTCAAGGTTTTTC | GATTACGCCAAGCTTTTAGTTTAAAAAATGGGAGC |
| *AsFAD2-15* | GATTACGCCAAGCTTACACTGGTATGCTTGCTCCTTA | GATTACGCCAAGCTTTGTCCCTTGTTGTAGTTCTCAC |
| *AsFAD2-16* | GATTACGCCAAGCTTTACAAGAGCCAGCCTACAGT | GATTACGCCAAGCTTCAGTGCGGTGGTATGGCTTTC |
| *AsFAD2-19* | GATTACGCCAAGCTTAGTAGAAGGGAAGGTGGTAG | GATTACGCCAAGCTTGAACCAGTAGACACCTTTGC |
| *AsFAD2-20* | GATTACGCCAAGCTTTTGAGCCAAGCACAACCCTTAT | GATTACGCCAAGCTTTGGTGCTTGCTACCTTCATCCG |
| *AsFAD2-21* | GATTACGCCAAGCTTCACTCCTTCATTTCCCTCCA | GATTACGCCAAGCTTAACCAAGGAACTCCATAGACAT |
| *AsFAD2-22* | GATTACGCCAAGCTTCCTCACCCTCTTCCTTACCTTGC | GATTACGCCAAGCTTCATTCCGCACTCTTCACCCCTTAT |
| *AsFAD2-23* | GATTACGCCAAGCTTCATCATGCTTACGCTCGGTTG | GATTACGCCAAGCTTGTGAACCATTATCAACCATCA |
| *AsFAD2-24* | GATTACGCCAAGCTTTGACAACCAAGAACCCATACAC | GATTACGCCAAGCTTAGCATTTAGGCTGGACTTCTTT |

Note: Underlined bases were added according to the instructions of SMARTer^®^ RACE 5′/3′ Kit.
