# Supplementary material for: Cloning and functional analysis of the FAD2 gene family from desert shrub Artemisia sphaerocephala
Source: BMC Plant Biol. 2019 Nov 8;19:481. doi: 10.1186/s12870-019-2083-5 (PMC6839233; doi:10.1186/s12870-019-2083-5)
Supplement: Supplementary file 10 — Additional file 10: Table S7. Primers used for amplification of the ORF of sixteen AsFAD2 genes in A. sphaerocephala. [file 12870_2019_2083_MOESM10_ESM.docx]

Table S7. Primers used for amplification of the ORF of sixteen *AsFAD2* genes.

| Primer gene | Sense sequence | Antisense sequence |
| --- | --- | --- |
| *AsFAD2-1* | ATGGGAGGAGGCGGGTGCAATG | TTACATCTTATTCTTGTACCAA |
| *AsFAD2-2* | ATGGTATCGGGCGGCTCTGCAA | TCACAGCTTATTTTTGAACCAA |
| *AsFAD2-4* | ATGGGAGCATCCGACGACATG | TTACTTTTTGAACCAGTAGACA |
| *AsFAD2-5* | ATGGGTGCGGGCGGGCATGCGAT | TTACATCTTATTGTTGAACCAGT |
| *AsFAD2-6* | ATGGGTTCGGGCGGCCGTGCA | TTACATCTTATTGTTGAACCA |
| *AsFAD2-7* | ATGGGAGCCGGTGGCCGGATGTC | TCACAACTTATGGTACCAATATA |
| *AsFAD2-8* | ATGGTAGCAACTGATGACTTGA | TTACTTTCTGAACCAATAAACA |
| *AsFAD2-9* | ATGGGCGCCGGTGGTCGTTCA | TCAATACTTATTGCTGTACCA |
| *AsFAD2-10* | ATGGGTGCAGGTGGACGAATGT | TCAGACCTTGTTACGGTACCAG |
| *AsFAD2-11* | ATGGGAGCTGGTGGCCAAATG | CTATTTGGAGAACCAATAAAC |
| *AsFAD2-13* | ACCTAGCCAAAGAGATCAAACA | CACAGCTTTGCGGACATTACAT |
| *AsFAD2-15* | ATGGGTTCTGGTGGCCGTGCT | TCACATTTTATTGTTGAACCA |
| *AsFAD2-20* | ATGGGTGCAGGTGGTCGAATGT | TTACATTTTGTGGTACCAGTAC |
| *AsFAD2-21* | ATGGGAGCATCCGACGACATGA | TTACTTTTTGAACCAGTAGACA |
| *AsFAD2-22* | ATGGGAGCAGGCGGTCGAATGT | TCACATTTTGTGGTACCAGTAT |
| *AsFAD2-23* | ATGTCGAAAACTGCGACTTTGAC | TTACTTTGGTAAGAACCA |
