# Supplementary material for: Cloning and functional analysis of the FAD2 gene family from desert shrub Artemisia sphaerocephala
Source: BMC Plant Biol. 2019 Nov 8;19:481. doi: 10.1186/s12870-019-2083-5 (PMC6839233; doi:10.1186/s12870-019-2083-5)
Supplement: Supplementary file 11 — Additional file 11: Table S8. Primers used for qRT-PCR study of sixteen AsFAD2 genes in A. sphaerocephala. [file 12870_2019_2083_MOESM11_ESM.docx]

Table S8. Primers used for qRT-PCR study of sixteen *AsFAD2* genes in *A. sphaerocephala*.

| Primer gene | Sense sequence | Antisense sequence |
| --- | --- | --- |
| *AsFAD2-1* | CATCGCCGCCACCACTCTAAC | GCCAGCCAAGTGTGAGAGTAGC |
| *AsFAD2-2* | ACCATCTTCGCAACCACCATTCAC | AGGCCAGGCCACGTACATAAGG |
| *AsFAD2-4* | ACCGAGTGGACTTGGATGAGAGG | TGCATGGTAGCGTGGAATAACAGG |
| *AsFAD2-5* | CGGACTTGTTGTAGCAGCCTATGG | ACCAAGTGATGACCACAAGGAACC |
| *AsFAD2-6* | ACCGAGTGGAACTGGCTGAGAG | TGGTGAGCAACATGCGTGTCTG |
| *AsFAD2-7* | TCATCGAAGTCACCATGCCAACAC | GTGAACACTCGACCAGGTGGATTG |
| *AsFAD2-8* | CCACGATTGCAGCCACCAT | TCACGGTACAAGCGATCTGAC |
| *AsFAD2-9* | ACTTGGCTTGAAGACTCTGTAGGC | GCGATCAAGTGAACCACAGTTAGC |
| *AsFAD2-10* | CGGTATCCTAGCTGTCATGTTCGG | CAGTTGAGTCGTAGTGAGGCAGTG |
| *AsFAD2-11* | GCCTCATTATCACGCCGTCGAG | GTGCTCCGATGAATCGTCAGGTTC |
| *AsFAD2-13* | GGTGCAGGTGGTCGAATGTCTG | GAACGGATGACAGATCGCTCGAAG |
| *AsFAD2-15* | CTCGTGGCAAGCTATGGTGTCTAC | GGCAGTGAGCGGTGAGTATGATG |
| *AsFAD2-20* | GCAGGTGGTCGAATGTCGGATAC | GGCAACAATGAGATCGTGGACAAC |
| *AsFAD2-21* | ACCGAGTGGACTTGGATGAGAGG | TGTGCATGGTAGCGTGGAATGG |
| *AsFAD2-22* | GGAGCAGGCGGTCGAATGTC | AACGAGAACGGTGGATCAACTGG |
| *AsFAD2-23* | CTCTCACCGTCGCCATCATTCG | GCTCCTCCTTAGTCGCCAATGTTG |
| *Actin* | CGAGACCACATACAACTCCATC | TGATTTCCTTGCTCATCCTG |

Note: The actin gene was used as an internal control.
