# Supplementary material for: Cloning and functional analysis of the FAD2 gene family from desert shrub Artemisia sphaerocephala
Source: BMC Plant Biol. 2019 Nov 8;19:481. doi: 10.1186/s12870-019-2083-5 (PMC6839233; doi:10.1186/s12870-019-2083-5)
Supplement: Supplementary file 12 — Additional file 12: Table S9. Primers used in subcellular localization study of seven AsFAD2 genes. [file 12870_2019_2083_MOESM12_ESM.docx]

Table S9. Primers used in subcellular localization study of seven *AsFAD2* genes.

| Primer gene | Sense sequence | Antisense sequence |
| --- | --- | --- |
| *AsFAD2-1* | ACGAACGATACTCGAGATGGGAGGAGGCGGGTGC | TCACTAGTACGTCGACCATCTTATTCTTGTACCAAAACAC |
| *AsFAD2-9* | ACGAACGATACTCGAGATGGGCGCCGGTGGTCGT | TCACTAGTACGTCGACATACTTATTGCTGTACCAATAG |
| *AsFAD2-10* | ACGAACGATACTCGAGATGGGTGCAGGTGGACGA | TCACTAGTACGTCGACGACCTTGTTACGGTACCAGTAG |
| *AsFAD2-11* | ACGAACGATACTCGAGATGGGAGCTGGTGGCCAAATG | TCACTAGTACGTCGACTTTGGAGAACCAATAAACACCT |
| *AsFAD2-15* | ACGAACGATACTCGAGATGGGTTCTGGTGGCCGTGCT | TCACTAGTACGTCGACCATTTTATTGTTGAACCAATA |
| *AsFAD2-20* | ACGAACGATACTCGAGATGGGTGCAGGTGGTCGA | TCACTAGTACGTCGACCATTTTGTGGTACCAGTACA |
| *AsFAD2-23* | ACGAACGATACTCGAGATGTCGAAAACTGCGACTTTGAC | TCACTAGTACGTCGACTTACTTTGGTAAGAACCA |

Note: Underlined bases were added according to the instructions of In-Fusion® HD Cloning Kit.
