# Supplementary material for: Cloning and functional analysis of the FAD2 gene family from desert shrub Artemisia sphaerocephala
Source: BMC Plant Biol. 2019 Nov 8;19:481. doi: 10.1186/s12870-019-2083-5 (PMC6839233; doi:10.1186/s12870-019-2083-5)
Supplement: Supplementary file 13 — Additional file 13: Table S10. Primers carrying restriction endonuclease used in heterologous expression of sixteen AsFAD2 genes in Saccharomy cescerevisiae. [file 12870_2019_2083_MOESM13_ESM.docx]

Table S10. Primers carrying restriction endonuclease used in heterologous expression of sixteen *AsFAD2* genes in *Saccharomyces cerevisiae.*

| Primer gene | Sense sequence | Antisense sequence |
| --- | --- | --- |
| *AsFAD2-1* | CATGGGTACCATGGGAGGAGGCGGGTGCAAT (KpnI) | CATGGAATTCTTAATGATGATGATGATGATGCATCTTATTCTTGTACCAA (EcoRI) |
| *AsFAD2-2* | CATGGGTACCATGGTATCGGGCGGCTCTGCA (KpnI) | CATGGAATTCTTAATGATGATGATGATGATGCAGCTTATTTTTGAACCAATA (EcoRI) |
| *AsFAD2-4* | CATGGGTACCATGGGAGCATCCGACGA (KpnI) | CATGCTCGAGTTAATGATGATGATGATGATGCTTTTTGAACCAGTAGACAC (XhoI) |
| *AsFAD2-5* | CATGGGTACCATGGGTGCGGGCGGG (KpnI) | CATGGAATTCTTAATGATGATGATGATGATGCATCTTATTGTTGAACCAGT (EcoRI) |
| *AsFAD2-6* | CATGGGTACCATGGGTTCGGGCGGCCGTGCA (KpnI) | CATGCTCGAGTTAATGATGATGATGATGATGCATCTTATTGTTGAACCAATA (XhoI) |
| *AsFAD2-7* | CATGGGATCCATGGGAGCCGGTGGCCGGATG (BamHI) | CATGCTCGAGTTAATGATGATGATGATGATGCAACTTATGGTACCAATATAC (XhoI) |
| *AsFAD2-8* | CATGGGTACCATGGTAGCAACTGATGACTTGA (KpnI) | CATGCTCGAGTTAATGATGATGATGATGATGCTTTCTGAACCAATAAACA (XhoI) |
| *AsFAD2-9* | CATGGGTACCATGGGCGCCGGTGGTC (KpnI) | CATGGAATTCTCAATGATGATGATGATGATGATACTTATTGCTGTACCAATAG (EcoRI) |
| *AsFAD2-10* | CATGAAGCTTATGGGTGCAGGTGGACGA (HindIII) | CATGGAATTCTCAATGATGATGATGATGATGGACCTTGTTACGGTACCAG (EcoRI) |
| *AsFAD2-11* | CATGGGTACCATGGGAGCTGGTGGCCA (KpnI) | CATGCTCGAGCTAATGATGATGATGATGATGTTTGGAGAACCAATAAACAC (XhoI) |
| *AsFAD2-13* | CATGGGATCCATGGGTGCAGGTGGTCGAA (BamHI) | CATGCTCGAGTTAATGATGATGATGATGATGCATTTTATGGTACCAGTATACA (XhoI) |
| *AsFAD2-15* | CATGGGTACCATGGGTTCTGGTGGCCG (KpnI) | CATGGAATTCTCAATGATGATGATGATGATGCATTTTATTGTTGAACCAATAAA (EcoRI) |
| *AsFAD2-20* | CATGGAGCTCATGGGTGCAGGTGGTCGA (SacI) | CATGGAATTCTTAATGATGATGATGATGATGCATTTTGTGGTACCAGTACA (EcoRI) |
| *AsFAD2-21* | CATGGGTACCATGGGAGCATCCGACGACATGA (KpnI) | CATGCTCGAGTTAATGATGATGATGATGATGCTTTTTGAACCAGTAGACA (XhoI) |
| *AsFAD2-22* | CATGGGATCCATGGGAGCAGGCGGTCGAATG (BamHI) | CATGCTCGAGTTAATGATGATGATGATGATGCATTTTGTGGTACCAGTATGT (XhoI) |
| *AsFAD2-23* | CATGGGATCCATGTCGAAAACTGCGACTTTG (BamHI) | CATGCTCGAGTTAATGATGATGATGATGATGCTTTGGTAAGAACCAATGAA (XhoI) |

Note: The underlined sequences are the corresponding restriction endonuclease.
